# Social Sector Expenditure and Child Mortality in India: A State-Level Analysis from 1997 to 2009

**DOI:** 10.1371/journal.pone.0056285

**Published:** 2013-02-07

**Authors:** Susanna M. Makela, Rakhi Dandona, T. R. Dilip, Lalit Dandona

**Affiliations:** 1 Public Health Foundation of India, New Delhi, India; 2 Institute for Health Metrics and Evaluation, University of Washington, Seattle, Washington, United States of America; Aga Khan University, Pakistan

## Abstract

**Background:**

India is unlikely to meet the Millennium Development Goal for child mortality. As public policy impacts child mortality, we assessed the association of social sector expenditure with child mortality in India.

**Methods and Findings:**

Mixed-effects regression models were used to assess the relationship of state-level overall social sector expenditure and its major components (health, health-related, education, and other) with mortality by sex among infants and children aged 1–4 years from 1997 to 2009, adjusting for potential confounders. Counterfactual models were constructed to estimate deaths averted due to overall social sector increases since 1997. Increases in per capita overall social sector expenditure were slightly higher in less developed than in more developed states from 1997 to 2009 (2.4-fold versus 2-fold), but the level of expenditure remained 36% lower in the former in 2009. Increase in public expenditure on health was not significantly associated with mortality reduction in infants or at ages 1–4 years, but a 10% increase in health-related public expenditure was associated with a 3.6% mortality reduction (95% confidence interval 0.2–6.9%) in 1–4 years old boys. A 10% increase in overall social sector expenditure was associated with a mortality reduction in both boys (6.8%, 3.5–10.0%) and girls (4.1%, 0.8–7.5%) aged 1–4 years. We estimated 119,807 (95% uncertainty interval 53,409 – 214,662) averted deaths in boys aged 1–4 years and 94,037 (14,725 – 206,684) in girls in India in 2009 that could be attributed to increases in overall social sector expenditure since 1997.

**Conclusions:**

Further reduction in child mortality in India would be facilitated if policymakers give high priority to the social sector as a whole for resource allocation in the country’s 5-year plan for 2012–2017, as public expenditure on health alone has not had major impact on reducing child mortality.

## Introduction

Despite over two decades of public programmes focusing on child health issues, child survival remains an area of concern in India. Available data show that although the under-five mortality rate has declined from 192 to 63 deaths per 1000 live births from 1970 to 2009 [1], [2], India still accounts for nearly a quarter of the 7.2 million under-five deaths globally every year [3]. A considerable gap remains in the way of achieving the Millennium Development Goal (MDG) 4 of reducing the under-five mortality rate to 39 deaths per 1000 live births by the year 2015 [3].

A recent call to action for universal health care in India has highlighted that the health gains in India over the last decade remain inadequate and are not commensurate with economic growth and increased political commitment to the social sector [4]. Poor child health outcomes in India are attributable to a mix of social determinants of health, including maternal education, socio-economic status, and access to health care [5]–[15]. The overall risk of child mortality is higher among female than in male children [2], [16].

The coverage of any of the public interventions for reproductive health, child health and nutrition is less than 55% in India [5]. In addition, public expenditure on education and health, which in 2009 constituted nearly 60% of state-level social sector expenditure in India [17], lags behind that of the other BRICS (Brazil, Russia, India, China, South Africa) countries [18]. This trend is not new, as even in the 1990s India spent less on the social sector as a proportion of its gross domestic product than many developing countries [19].

Public expenditure on health has been reported in the past to have a variable relationship with health outcomes in India. Farahani et al used cross-sectional data from Indian states at a single time point in 1998–99 to suggest that increased public expenditure on health reduced the probability of death across all ages [20], while Bhalotra found a significant inverse relationship between state-level health expenditure and infant mortality only in rural areas using a time series of mortality and expenditure from 1970 to 1998 [21]. A third study in India examined the relationship between public expenditure on health and infant mortality using data spanning 1980 to 1999 and concluded that although there was some evidence of a negative association between the two, the results were not robust to alternative model specifications [22].

The effect of public expenditure on health outcomes in other developing contexts has also been examined by several studies. Public health expenditure has been found to be significantly associated with improved health status in Lesotho [23] and Central America and the Caribbean [24]. A 2005 study of mortality levels and trends in China in the 1980s and 1990s found that public expenditure on education and health was significantly associated with increased life expectancy at birth [25]. Cross-country studies of developing countries [26], [27] and transitioning economies [28] have also found evidence for a negative relationship between public expenditure on health and infant and child mortality. However, evidence on the relationship between overall social sector expenditure and health outcomes in developing countries remains scarce, particularly in South Asia.

Given the likely role of broader social determinants of health on child health outcomes and the mixed previous evidence on the effect of public expenditure on health in India, we examined the association of overall social sector expenditure and its major components with child mortality in India from 1997 to 2009.

## Methods

We investigated the association of state-level health, health-related, education, other social sector, and overall social sector expenditure with mortality by sex for infants and children aged 1–4 years in India between 1997 and 2009, controlling for potential confounders.

### Social sector expenditure

Data on state-level expenditure in all sectors are available from the Reserve Bank of India (RBI) [17]. For this analysis, social sector expenditure was divided in the following categories: health, health-related, education, and other. Health expenditure included spending on medical and public health and family welfare. Health-related expenditure included spending on health, nutrition, water supply, and sanitation. Education expenditure included spending on education, sports, art, and culture (as reported by RBI); education accounts for about 95% of the expenditure in this category [29]. Other social sector expenditure included spending on housing, urban development, welfare of scheduled castes/tribes and other backward castes, labour and labour welfare, social security and welfare, relief on account of natural calamities, and other miscellaneous social sector categories. Overall social sector expenditure included all of the preceding categories. We used actual expenditure estimates for the fiscal years 1997 through 2009. Table S1 in File S1 lists the composition of overall social sector expenditure in terms of health, nutrition, water supply and sanitation, education, and other overall social sector expenditure.

The state-level social sector expenditure data include funds from the federal government for various national public health programmes, which comprise about 20% of the total state social sector expenditure on average. Funding allocation for states for some such recent central government schemes, representing a small proportion of the total, was not available and is not reflected in the state-level expenditure used in this analysis.

For comparison of expenditure over time, we converted budgetary expenditure data from current prices to constant prices using the net state domestic product (NSDP) deflator with the 2009 fiscal year as the base year [17]. We calculated the average expenditure for the five years ending in the index year to account for the cumulative impact of spending on the under-5 population. Population data were single-year census-based projections, the details of which are presented in Text S1.

### Mortality data

We obtained mortality data by sex for infants and children aged 1–4 years from 1997 to 2009 from the Sample Registration System (SRS) of the Office of the Registrar General of India [2]. In the current sampling frame, SRS has continuous registration of births and deaths in 7597 randomly chosen enumeration areas (4433 rural, 3164 urban) covering nearly 7.2 million people and produces demographic data representative of the rural and urban areas of each state in India [2]. Deaths per 1000 population (death rates) were considered for the 1–4 years age group, and deaths per 1000 live births (mortality rates) were considered for infants. Published data from the SRS are available for 20 major states and union territories of India [2]. Nineteen of these states, which comprised 96.5% of the population of India in 2011 [30], were included in this analysis: Andhra Pradesh, Assam, Bihar, Chhattisgarh, Delhi, Gujarat, Haryana, Himachal Pradesh, Jharkhand, Karnataka, Kerala, Madhya Pradesh, Maharashtra, Odisha, Punjab, Rajasthan, Tamil Nadu, Uttar Pradesh, and West Bengal. Data from the state of Jammu and Kashmir were not used due to non-availability of child mortality data for a substantial part of the study period.

Of the states for which the SRS provides death rate data, two were created in the year 2000: Jharkhand from Bihar and Chhattisgarh from Madhya Pradesh. These new states are quite populous, accounting for roughly 25% of the population in their parent states [30]. To generate comparable time series from 1997 to 2009, we combined data for Bihar with Jharkhand and for Madhya Pradesh with Chhattisgarh. Additionally, a small state Uttarakhand was split off from Uttar Pradesh in 2001, comprising about 5% of the population of the parent state. SRS does not report death rates for small states like Uttarakhand. We therefore use data for Uttar Pradesh as reported by the SRS from 1997 to 2009.

The states of Bihar, Chhattisgarh, Jharkhand, Madhya Pradesh, Odisha, Rajasthan, Uttarakhand, and Uttar Pradesh in northern and central India are relatively less developed and are referred to as the Empowered Action Group (EAG) states in India [31]. The north-eastern states of India are also relatively less developed, and SRS reports data on Assam, which is the largest state in this group. In this paper, we report data for Assam along with the EAG states and refer to them as less developed states. We refer to the other states for which we use data reported by the SRS as more developed states; these include Andhra Pradesh, Delhi, Gujarat, Haryana, Himachal Pradesh, Karnataka, Kerala, Maharashtra, Punjab, Tamil Nadu, and West Bengal.

### Effect of state-level expenditure on child mortality

We investigated the relationship between state-level health, health-related, education, other social sector, and overall social sector expenditure and child mortality from 1997 to 2009 while controlling for potential confounders. We modelled the natural log of the sex-specific infant mortality rate and death rate at ages 1–4 as a function of the natural log of per-capita expenditure while controlling for poverty rates:
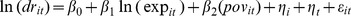



where *dr_it_* is the death or mortality rate in year *t* and state *i*, *exp_it_* is per-capita health, health-related, education, other, or overall social sector expenditure, and *pov_it_* is the poverty rate. The intercept is given by *β_0_*, while *β_1_* represents the elasticity of mortality to expenditure – in other words, the percent change in mortality due to a 1% change in per-capita expenditure. When multiplied by 100, *β_2_* represents the percent change in mortality due to a one percentage-point change in poverty rates. The error term is given by *ε_it_*. The model was run separately for each of the four age-sex groups (infants and ages 1–4, boys and girls) to account for the differing determinants of mortality at these ages and differing patterns by sex in India.

We included poverty rates (*pov_it_*) because substantial economic growth may not be evenly distributed in a population and may not necessarily translate into lower poverty rates. Poverty estimates were available from the Planning Commission of India for the years 1973–74, 1977–78, 1983, 1987–88, 1993–94, 1999–2000, and 2004–05 [17]. We regressed log poverty rates on year in each state and then predicted annual poverty rates from 1997 to 2009 to generate a complete time-series for our years of interest, using the natural log to avoid predicting negative poverty rates.

The model includes a random intercept by state (*η_i_*) to capture state-specific factors affecting child mortality and a random intercept by year (*η_t_*) to account for changes over time that affect all states. We did not use a fixed effect on time because the regression for estimating poverty rates already uses such a fixed effect. While many factors affect child mortality, we included poverty in our model because it is a social determinant that influences many other factors that impact health status. The effects of any omitted explanatory variables at the state level in our model would be accounted for by the state random intercept.

We ran separate models for each component of social sector expenditure instead of including them as separate variables in a single model due to the high correlations between the components (Table S2 in File S1). We also investigated alternative measures of state-level social sector expenditure as a percentage of total state expenditure and as a percentage of state domestic product. However, we chose to use per-capita expenditure, as this measure is not influenced by the size of a state’s economy or funds spent in other sectors.

In addition, we investigated the association of expenditure on nutrition, water supply and sanitation as a category with child mortality. We explored women’s literacy, defined as the percentage of illiterate women aged 15–35 years, as a variable in the model both instead of and in addition to poverty. We also considered alternate models, including a first differences model, a model using indicator variables (“fixed effects”) for each state instead of random intercepts, and a model with a sex-expenditure interaction. Details of these alternate models are given in Text S2.

### Estimation of averted deaths

To assess the impact of state-level overall social sector expenditure, we computed counterfactual estimates of the number of child deaths that were averted between 1997 and 2009 under two different scenarios:

- if state-level overall social sector expenditure had remained at its 1997 levels and
- if poverty rates had remained at their 1997 levels.

We used the regression model described previously to predict state-level mortality and death rates from 1997 to 2009 for all age and sex groups. We calculated counterfactual mortality and death rates by setting first overall social sector expenditure and then poverty rates to their 1997 values.

Then, we calculated the difference between the observed and counterfactual mortality and death rates for the two scenarios, which we then multiplied by the population denominator for the corresponding age-sex group to estimate the number of deaths averted by state and year. The observed data come from the model predictions, which provide smoother trends over time than the raw mortality data. Uncertainty intervals for the number of averted deaths were calculated by simulation by taking 1000 draws from the uncertainty bounds of the estimated parameters (see Text S1 p3 for details). Annual estimates of population denominators by sex for our age groups of interest had to be indirectly calculated from available data. Details are presented in Text S1 (pp 1–2).

We used the sum of averted deaths at the state level as an estimate of the number of averted deaths at the national level. While the SRS only publishes mortality data for major states, these states comprise 96.5% of India's population in 2011 [30], and the aggregates are therefore representative of the national figures.

All analyses were done in Stata (version 11.2).

### Ethics Statement

An ethics statement was not required for this work.

## Results

### Trends in social sector expenditure

**Figure 1:**
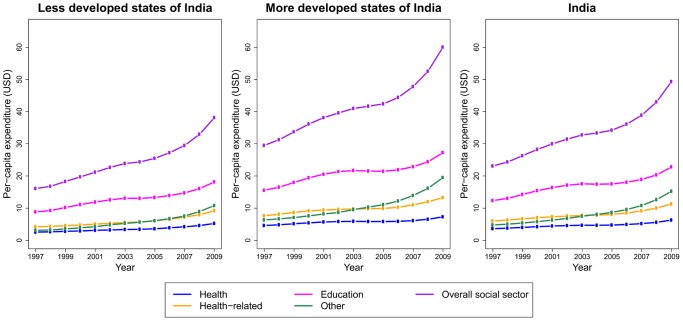
Per-capita health, health-related, education, other, and overall social sector expenditure, 1997–2009. Per-capita expenditure is calculated as the sum of yearly expenditure across all states in our analysis divided by the sum of yearly population in these states and averaged for the five years ending in the index year. Expenditure is shown in constant 2009 prices (USD). Trends are shown for the less developed and more developed state of India and for India as a whole.

Per-capita health expenditure at constant prices, averaged over the five years ending in the index year, increased 1.7-fold (range 1.3–3.2), health-related expenditure 1.9-fold (1.4–3.0), education 1.9-fold (1.3–3.0), other social sector expenditure 3.2-fold (2.0–6.7), and overall social sector expenditure 2.1-fold (1.5–3.6) across the Indian states between 1997 and 2009. These increases were mostly due to the accelerated expenditure since 2004 that followed a stagnation in the early 2000s (Figure 1). Education expenditure was the major component of social sector expenditure across states in both 1997 (53%) and 2009 (45%). Other social sector expenditure constituted 21% of overall social sector expenditure in 1997 and 33% in 2009. Health and health-related expenditure were relatively smaller, accounting for 16% and 26% of overall social sector expenditure in 1997 and 12% and 22% in 2009 (Table S1 in File S1).

In 2009, per-capita expenditure on the overall social sector in less developed states (INR 1808 [US$38]) was 64% of that in more developed states (INR 2845 [US$60]), despite relatively larger increases in the former group (2.4-fold [range 1.4–3.2]) than the latter (2.0-fold [1.5–2.3]) since 1997. The less developed states had lower expenditure than the more developed states in health (INR 248 [US$5] vs. INR 346 [US$7]), health-related (INR 436 [US$9] vs. INR 630 [US$13]), education (INR 860 [US$ 18] vs. INR 1292 [US$ 27]), and other social sector (INR 511 [US$ 11] vs. INR 924 [US$ 20]) in 2009 (Figure 1). The less developed state of Uttar Pradesh had the largest increases in per-capita health (3.2-fold), health-related (3.0-fold), education (3.0-fold), other social sector (6.7-fold), and overall social sector (3.6-fold) expenditure between 1997 and 2009. Despite these large increases, Uttar Pradesh continued to remain in the low expenditure end of the spectrum. By contrast, per-capita overall social sector expenditure in 2009 in the more developed state of Himachal Pradesh was 3.7-fold higher than in Uttar Pradesh, and health, health-related, education, and other social sector expenditures were 3.0, 6.5, 3.7, and 1.9 fold higher, respectively. State-level trends in social sector expenditure and poverty rates are shown in Figures S1 and S2.

### Mortality trends

**Figure 2:**
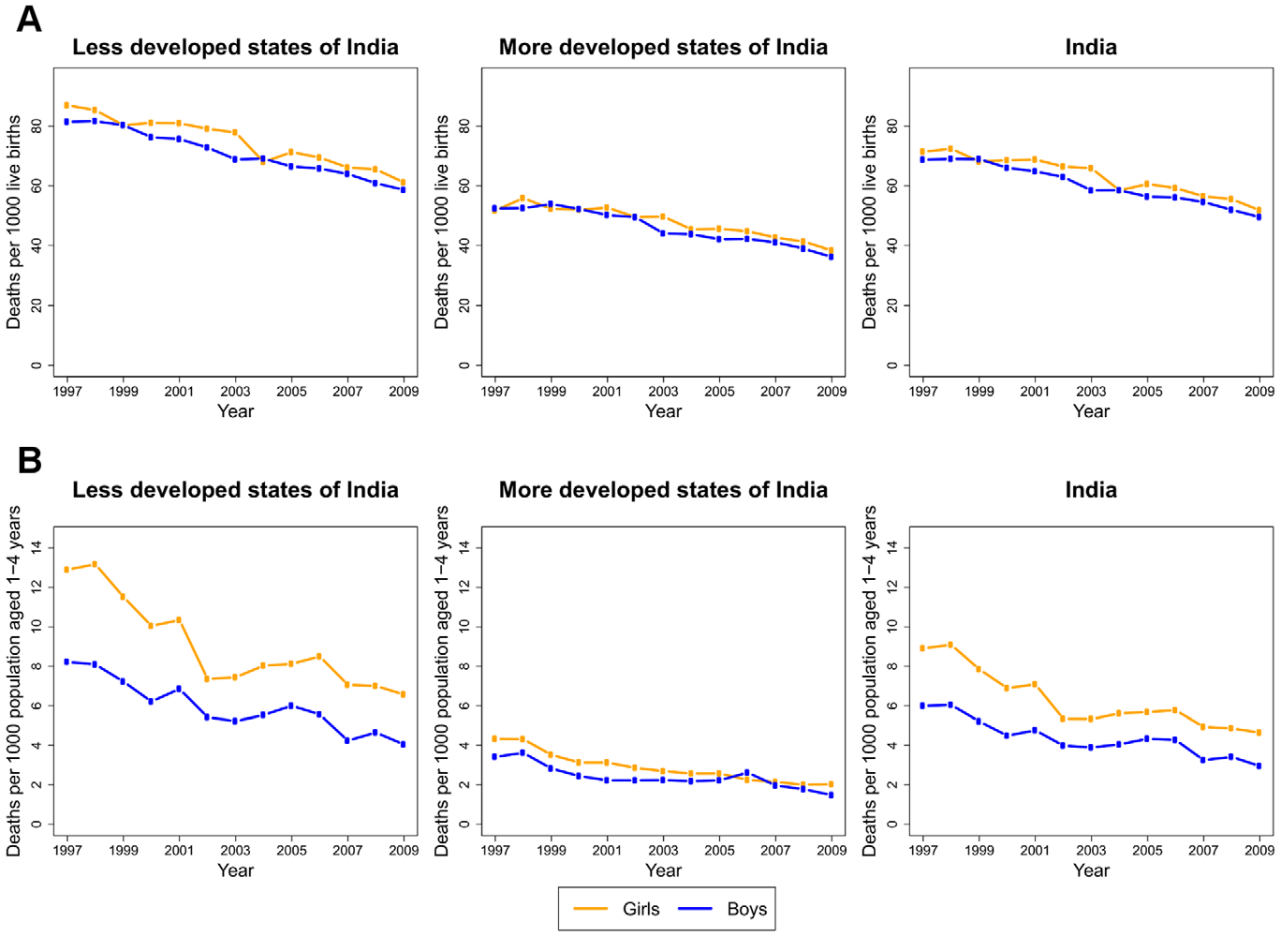
Mortality trends among A) infants and B) children aged 1–4 years, 1997–2009. Trends are shown for the less developed and more developed state of India and for India as a whole.

The infant mortality rate across states changed from 69 to 50 in boys and from 71 to 52 in girls between 1997 and 2009, declines of 28% and 29%, respectively (Figure 2). Death rates in children aged 1–4 years reduced by 51% in boys (6.0 to 3.0 per 1000 population) and 48% in girls (8.9 to 4.6 per 1000 population) during this period.

Percent declines in mortality from 1997 to 2009 were similar between the less developed and more developed states, but absolute mortality levels remained higher in the less developed states in 2009 (Figure 2). The death rate in girls aged 1–4 years was over three times larger in the less developed states than in the more developed states (6.6 vs. 2.0 per 1000 population), and that in boys aged 1–4 years was over twice in the less developed states than in the more developed states (4.0 vs. 1.5). Differentials in infant mortality in 2009 were smaller, with rates in the less developed states about 1.6-times those in the more developed states.

Death rates at ages 1–4 years declined sharply between 1997 and 2009 in several less developed states (Figure S3). In Bihar, death rates in 2009 were less than half of their 1997 levels in girls (5.9 vs. 12.6 per 1000 population) and boys (3.7 vs. 8.2). Death rates in boys were over 3.5-fold smaller in Rajasthan in 2009 than in 1997 (2.2 vs. 7.8). Proportional reductions in infant mortality were relatively smaller for both less developed and more developed states, with the largest drops between 1997 and 2009 in girls in Tamil Nadu (57 to 29 per 1000 live births) and boys in Maharashtra (52 to 28) (Figure S4).

The gender gap in mortality, defined as the ratio of mortality in girls to that in boys, increased in both less developed and more developed states for ages 1–4 between 1997 and 2009. This increase was relatively larger in the more developed states, from 1.27 in 1997 to 1.37 in 2009, as compared with the less developed states (1.57 to 1.62). The gender gap in infant mortality increased between 1997 and 2009 in the less developed states (0.99 to 1.06) but decreased slightly in the more developed states (1.07 to 1.04).

### Relationship between social sector expenditure and child mortality

We did not find evidence of a statistically significant relationship between per capita public expenditure on health and mortality for either infants or ages 1–4 years (Table 1). Adding expenditure on nutrition and water and sanitation to health expenditure made this relationship somewhat stronger, as it was statistically significant for boys aged 1–4 years but not for the other age-sex groups. Education expenditure had a negative relationship with mortality at ages 1–4 years, with a 10% increase in per-capita education expenditure associated with an 8.5% (95% confidence interval 4.9–12.0%) reduction in the death rate in boys and a 4.7% (1.1–8.3%) reduction in girls aged 1–4 years. There was no significant association between education expenditure and infant mortality. A 10% increase in per-capita other social sector expenditure was associated with decreased mortality in infant girls (1.1% [0.3 – 1.8%]) and in boys (2.4% [0.4–4.3%]) and girls (2.2% [0.4–4.1%]) aged 1–4 years.

**Table 1:** Association of infant mortality rate and death rate for age 1–4 years with per-capita state social sector expenditure from 1997 to 2009, using a mixed-effects regression model.

Age-sex group	Model term	Social sector expenditure component (95% CI)				
		Health	Health-related	Education	Other	Overall
Infants, boys						
	Expenditure	0.06 (−0.05 to 0.16)	0.07 (−0.03 to 0.18)	0.01 (−0.12 to 0.14)	0.01 (−0.06 to 0.08)	0.00 (−0.12 to 0.12)
	Poverty	0.05 (0.04 to 0.06)^a^	0.05 (0.04 to 0.06)^a^	0.05 (0.04 to 0.05)^a^	0.05 (0.04 to 0.06)^a^	0.05 (0.03 to 0.06)^a^
	Intercept	2.64 (1.91 to 3.37)^a^	2.49 (1.67 to 3.32)^a^	2.94 (1.90 to 3.98)^a^	2.95 (2.33 to 3.58)^a^	3.00 (1.88 to 4.12)^a^
	σ_t_ (year)	0.03 (0.01 to 0.05)^a^	0.03 (0.01 to 0.06)^a^	0.02 (0.01 to 0.05)^a^	0.03 (0.01 to 0.05)^a^	0.02 (0.01 to 0.05)^a^
	σ_i_ (state)	0.49 (0.34 to 0.70)^a^	0.49 (0.34 to 0.70)^a^	0.49 (0.34 to 0.70)^a^	0.49 (0.34 to 0.71)^a^	0.49 (0.34 to 0.70)^a^
Infants, girls						
	Expenditure	0.10 (0.03 to 0.05)	0.01 (−0.12 to 0.13)	0.03 (−0.12 to 0.18)	−0.11 (−0.18 to −0.03)^a^	−0.08 (−0.22 to 0.06)
	Poverty	0.04 (0.03 to 0.05)^a^	0.04 (0.03 to 0.05)^a^	0.04 (0.03 to 0.05)^a^	0.03 (0.01 to 0.04)^a^	0.03 (0.02 to 0.04)^a^
	Intercept	2.58 (1.71 to 3.44)^a^	3.20 (2.22 to 4.17)^a^	3.01 (1.77 to 4.24)^a^	4.09 (3.41 to 4.77)^a^	3.92 (2.63 to 5.21)^a^
	σ_t_ (year)	0.03 (0.01 to 0.07)^a^	0.03 (0.01 to 0.07)^a^	0.03 (0.01 to 0.07)^a^	0.03 (0.00 to 0.08)^a^	0.02 (0.01 to 0.06)^a^
	σ_i_ (state)	0.47 (0.33 to 0.67)^a^	0.46 (0.32 to 0.66)^a^	0.46 (0.32 to 0.67)^a^	0.42 (0.30 to 0.60)^a^	0.44 (0.31 to 0.64)^a^
1–4 years, boys						
	Expenditure	−0.31 (−0.66 to 0.05)	−0.36 (−0.69 to −0.02)^a^	−0.85 (−1.20 to −0.49)^a^	−0.24 (−0.43 to −0.04)^a^	−0.68 (−1.00 to −0.35)^a^
	Poverty	0.04 (0.02 to 0.06)^a^	0.04 (0.02 to 0.06)^a^	0.03 (0.01 to 0.05)^a^	0.04 (0.02 to 0.06)^a^	0.03 (0.00 to 0.05)^a^
	Intercept	1.91 (−0.38 to 4.19)	2.42 (−0.00 to 4.84)	6.27 (3.49 to 9.05)^a^	1.66 (0.18 to 3.13)^a^	5.59 (2.77 to 8.42)^a^
	σ_t_ (year)	0.09 (0.04 to 0.22)^a^	0.08 (0.03 to 0.22)^a^	0.02 (0.00 to 14.30)	0.08 (0.03 to 0.19)^a^	0.04 (0.01 to 0.28)^a^
	σ_i_ (state)	0.48 (0.33 to 0.72)^a^	0.51 (0.35 to 0.74)^a^	0.46 (0.32 to 0.66)^a^	0.49 (0.34 to 0.70)^a^	0.46 (0.32 to 0.67)^a^
1–4 years, girls						
	Expenditure	0.03 (−0.30 to 0.36)	−0.04 (−0.36 to 0.28)	−0.47 (−0.83 to −0.11)^a^	−0.22 (−0.41 to −0.04)^a^	−0.41 (−0.75 to −0.08)^a^
	Poverty	0.08 (0.06 to 0.10)^a^	0.08 (0.05 to 0.10)^a^	0.06 (0.03 to 0.08)^a^	0.05 (0.03 to 0.08)^a^	0.05 (0.02 to 0.08)^a^
	Intercept	−0.51 (−2.69 to 1.66)	−0.04 (−2.42 to 2.35)	3.37 (0.50 to 6.24)^a^	1.54 (−0.00 to 3.08)	3.38 (0.39 to 6.36)^a^
	σ_t_ (year)	0.05 (0.01 to 0.21)^a^	0.05 (0.01 to 0.20)^a^	0.03 (0.00 to 0.62)^a^	0.05 (0.02 to 0.16)^a^	0.04 (0.01 to 0.19)^a^
	σ_i_ (state)	0.81 (0.55 to 1.20)	0.80 (0.54 to 1.18)	0.70 (0.47 to 1.03)	0.67 (0.46 to 0.99)^a^	0.68 (0.46 to 1.00)

Expenditure is measured in log per-capita terms and averaged for the five years ending in the index year. 95% confidence intervals are given in parentheses.

a Statistically significant coefficient (5% level)

Increased per-capita state overall social sector expenditure was associated with a decrease in mortality for both boys and girls aged 1–4 years (Table 1). In boys aged 1–4 years, an increase of 10% in per-capita overall social sector expenditure was associated with a 6.8% (95% confidence interval 3.5–10.0%) reduction in death rate, while for girls the corresponding reduction was 4.1% (0.1–7.5%). With a 10% reduction in poverty rate, the associated reduction in death rates for boys and girls aged 1–4 years was 25% (2–48%) and 51% (24–77%), respectively.

Overall social sector expenditure was not significantly associated with infant mortality in either boys or girls. A 10% reduction in poverty was associated with a 45% (95% confidence interval 34–55%) and 31% (19–43%) reduction in infant mortality rates for boys and girls, respectively.

After controlling for poverty and overall social sector expenditure, the residual variation across states in the model was substantially larger than the residual variation across years (Table 1, File S2); the same was true for the models for separate components of social sector expenditure.

### Averted deaths

We estimated 1.31 million deaths in infants and 339,000 deaths in children aged 1–4 years across the major Indian states in 2009, down respectively from 1.76 million and 694,000 deaths in 1997. In 2009, 648,552 (49.4%) of the infant deaths were in girls, while 197,638 (58.4%) of deaths at ages 1–4 years were in girls. Had overall social sector expenditure remained at 1997 levels, we estimate that in 2009 there would have been 260,655 (95% uncertainty interval 190,800–372,618) deaths in boys aged 1–4 and 291,675 (188,903–447,380) deaths in girls aged 1–4, indicating that overall social expenditure reduced the total potential deaths in boys by 46% and in girls by 32% (Table 2). Over 80% of averted deaths attributed to increased overall social sector expenditure in ages 1–4 years in 2009 were in the less developed states, despite the size of the population aged 1–4 years being nearly evenly split between the less developed and more developed states (54% vs. 46%). The number of deaths averted in infants by increases in overall social sector expenditure was not significantly different from zero, but poverty reduction had a substantial impact on reducing deaths in infants (Table 1). Averted deaths in 2009 by state for the two counterfactual scenarios are shown in Table B in Text S1.

**Table 2:** Observed, counterfactual, and averted deaths in 2009.

Sex	Age (years)	Observed deaths	Counterfactual deaths (95% UI)		Averted deaths (95% UI)	
			Overall social sector expenditure	Poverty	Overall social sector expenditure	Poverty
boys	<1	664,441	663,536 (525,847 – 875,148)	956,089 (743,058 – 1,305,265)	0 (0 – 77,258)	291,648 (192,599 – 450,952)
girls	<1	648,552	694,762 (554,677 – 909,632)	834,714 (654,699 – 1,097,702)	46,210 (0 – 147,538)	186,162 (100,372 – 306,422)
boys	1 to 4	140,848	260,655 (190,800 – 372,618)	173,579 (120,313 – 265,790)	119,807 (53,409 – 214,662)	32,731 (2,285 – 89,345)
girls	1 to 4	197,638	291,675 (188,903 – 447,380)	300,144 (177,411 – 520,209)	94,037 (14,725 – 206,684)	102,506 (35,118 – 231,906)

Counterfactual and averted deaths are estimated for the scenarios in which overall social sector expenditure and poverty rates would have remained at their 1997 levels. (UI  =  uncertainty interval)

**Figure 3:**
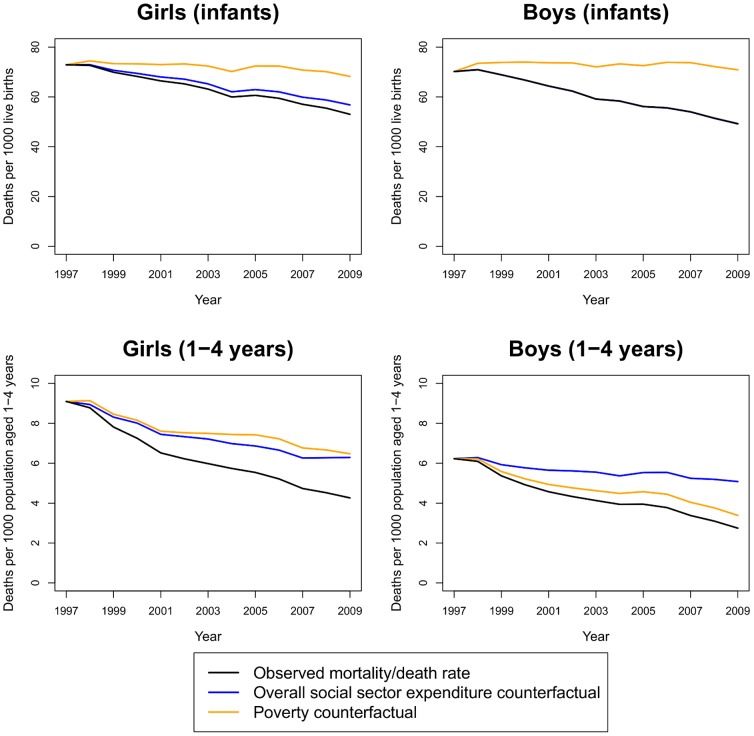
Observed and counterfactual mortality trends among infants and children aged 1–4 years, 1997–2009. The observed mortality rate (for infants) or death rate (for age 1–4) is shown in black; the overall social sector expenditure counterfactual shows the predicted mortality/death rates if state per-capita overall social sector expenditure had remained at its 1997 level (blue); the poverty counterfactual shows the predicted mortality/death rates if poverty rates had remained at their 1997 levels (orange). (Note: the effect of overall social sector expenditure on mortality is essentially zero in boys less than one. Hence, the mortality rate under the overall social sector expenditure counterfactual [blue] coincides with the estimated mortality rate [black].)

Observed and counterfactual death rates by age and sex at the national level are shown in Figure 3. Had overall social sector expenditure remained at the 1997 levels at constant prices, our results indicate that death rates for ages 1–4 years in 2009 would have been 85% higher for boys and 48% higher for girls. If poverty rates had remained at the 1997 levels, death rates in 2009 would have been 23% higher in boys and 52% higher in girls aged 1–4. While infant mortality rates were affected little by increased overall social sector expenditure, they would have been 22% higher in girls and 31% higher in boys in 2009 if poverty rates had remained at 1997 levels.

**Figure 4:**
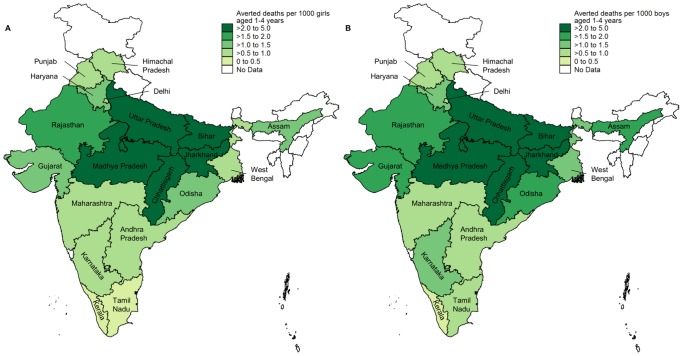
Averted deaths in 2009 by sex attributable to increased social sector spending since 1997. Averted deaths per 1,000 population among A) girls and B) boys aged 1–4 years. The population denominator is the sex-specific population aged 1–4 years in 2009. In the analysis, data from Jharkhand was combined with that from Bihar and data from Chhattisgarh with that from Madhya Pradesh. The averted death rates shown in the maps for these four states are therefore the averted death rates obtained for Bihar combined with Jharkhand and Madhya Pradesh combined with Chhattisgarh.

Figure 4 shows the number of averted deaths per 1000 population aged 1–4 years by state and sex in 2009 attributable to increases in overall social sector expenditure since 1997. The largest per capita gains in both boys and girls were in less developed states with high mortality in this age group: Uttar Pradesh, Madhya Pradesh including Chhattisgarh, and Bihar including Jharkhand. These per-capita gains were smaller in the more developed and wealthier states such as Himachal Pradesh, Kerala, Maharashtra, Punjab and Tamil Nadu. In the less developed states as a whole, averted deaths per unit population were over four times larger than in the more developed states for girls (3.1 vs. 0.7 deaths averted per 1000 girls aged 1–4 years) and over three times larger for boys (3.4 vs. 1.1 deaths averted per 1000 boys aged 1–4 years).

## Discussion

Our findings suggest that increases in overall state social sector expenditure have contributed to substantial reductions in mortality among children aged 1–4 years between 1997 and 2009 in India. We estimate that these increases in overall state social sector expenditure have averted about 214,000 deaths in India in children aged 1–4 years in 2009 – 120,000 in boys and 94,000 in girls. These reductions were larger in the less developed states with higher levels of mortality in 1997. The mortality however remains higher in the less developed states, indicating more scope for reducing it with further increases in social sector expenditure. It is important to note that the relationship between social sector expenditure and child mortality in a population is not static and depends in part on the existing level of child mortality; therefore, the effects of increased social sector expenditure may change with future reductions in child mortality.

We did not find evidence of a statistically significant relationship between overall social sector expenditure and infant mortality, possibly because mortality in this age group is dependent more on biological factors operating at the individual level than social factors. In addition, we did not find a significant association between public expenditure on health (13% of the total social sector expenditure in 2009) and mortality in infants or 1–4 years old children, suggesting that health expenditure alone has not been enough in impacting child mortality in India during 1997–2009. Previous reports from India using data up to 1999 have reported an ambiguous relation between public expenditure on health and infant mortality [20]–[22]. It is to be noted that public expenditure on health in India continues to be quite low, and major increases in public health expenditure have recently been suggested, with emphasis on comprehensive primary healthcare and universal health coverage [4], [32], [33]. If this were to happen, it is possible that the beneficial impact on child mortality may become observable.

However, we found that the addition of expenditure on nutrition, water supply and sanitation to health under health-related expenditure, which constituted 23% of the social sector expenditure in 2009, had a significant association with mortality reduction in boys 1–4 years of age during 1997–2009 but not for girls, suggesting the important role played by nutrition, water supply and sanitation. Expenditure on the education category, the largest component of social sector expenditure in India (45% in 2009), and on the other category comprising of the remaining social sector expenditure (31% in 2009), were associated with mortality reduction in both boys and girls 1–4 years of age. This association with education is supported by the known inverse relationship between maternal education and child mortality [34], [35] and the increasing educational attainment among women of childbearing age in India [36]. Our finding of the beneficial association with the other category, which includes housing, urban development, labour and other welfare activities, is plausible as these broader improvements can contribute to child mortality reduction. Overall, the increasingly strong gradient of the association between expenditure on health, health-related, and the overall social sector with reduction in mortality in 1–4 years old children indicates that while increases in public expenditure on health are important, larger benefits could accrue with increases in public expenditure in the broader social sector such as education, housing, urban development, and employment.

Poverty reduction had a larger impact on reducing infant mortality in boys and girls than did increases in social sector spending. The impact of increases in overall social sector spending was higher than that of poverty in 1–4 year old boys, and almost equal to that in 1–4 year old girls. Among the deaths in India under 5 years of age, 28% were in the 1–4 years age group in 1997 and 21% in 2009, and the remaining among infants (Table 2). Given these proportions, the overall impact of poverty reduction has been higher than that of increased social sector spending on reducing child mortality. However, significant changes in poverty rates are achievable only in the long term and require complex combinations of economic growth and government policies across multiple sectors, and such efforts must be effectively targeted toward those who are truly poor to have the desired impact. On the other hand, increasing social sector expenditure is relatively easier to accomplish in the short and medium term, as India has a 5-year planning cycle and budgets are set on a yearly basis. The two are also related to each other in an interactive manner. Both, therefore, should receive high priority in India.

The continuing higher death rates among girls than in boys, especially in the 1–4 years age group in the less developed states, are of concern. In addition, the coefficients of the beneficial impact of increases in public expenditure in the social sector as a whole or its components were higher for boys than for girls in our analysis. Particular attention will be needed in India to ensure that the benefits of increased social sector expenditure are not biased away from girls through preferential treatment of boys operating at household and community levels.

Although the rate of increase in per capita social sector expenditure has been slightly higher in the less developed states than in the more developed states of India during 1997–2009, the absolute level of expenditure per capita continues to be lower in the former. Coupled with the finding that per capita reductions in death rates among the 1–4 year olds attributable to increases in social sector spending were higher in the less developed states, there is a strong case for increasing further the social sector spending in these states. Besides facilitating reductions in child deaths, this would have broader societal benefits as well.

Our study has several limitations that should be considered when interpreting these results. First, this study uses data only at the state level. Ideally, we would have combined these data with individual- and household-level data in a multilevel model to better understand the interplay between the distal and proximal determinants of mortality, but the available individual-level data on mortality at ages 1–4 years across states are not suitable for this analysis. The National Family Health Surveys contain too few deaths in this age group to reliably identify the effects of state-level expenditure. The birth histories in the first and third rounds of the District Level Household Surveys (DLHS) in 1998–1999 and 2007–2008 preclude construction of long time series of mortality and analysis of the 1–4 years age group. The complete birth histories available in DLHS-2 (2002–2004) would restrict the analysis of this age group to the late 1990s and earlier. Given these limitations, we used publicly available data from SRS covering a 13-year time period instead of a single cross section, and our model specifications attempt to control for unexplained variation across states and years using random effects for these variables in the model, an approach that has been used in the literature previously [36]. It should also be noted that state-level data can mask considerable variations in and clustering of mortality rates within states. A recent district-level geospatial analysis of child mortality in India reported large within-state variations in district-level under-five mortality rates (e.g. 52 to 142 deaths per 1000 live births in Uttar Pradesh), with significant overlap in the clustering of high-mortality and low-health coverage districts [37]. While it would be useful to repeat the present study at the district level, available data do not allow for the disaggregation of expenditure data from the state to the district level.

Second, the SRS does not publish data from smaller states and union territories due to issues of sample size, and therefore our estimates of deaths averted at the national level are based on data from a subset of major Indian states. However, because this subset of states accounts for 96.5% of the national population [30], the missing states would not have a significant impact on our results. Third, population denominators used to calculate averted deaths are not directly available. We therefore computed these indirectly from available data as described in text S1 (pp 1–2), corrected for gaps and implausible fluctuations, to generate these population estimates. This is unlikely to have caused major biases in our findings. Finally, while we have found evidence for a statistically and practically significant relationship between overall social sector expenditure and mortality at ages 1–4 years, we are not able to analyze the pathways through which this effect may occur. More detailed research is required for this understanding which is beyond the scope of this paper.

Even with these limitations, this study contributes to the knowledge on the relationship between social sector public expenditure and health outcomes, as it examines the impact of each major component of the social sector expenditure, and overall social sector expenditure, on sex-specific mortality for infants and children aged 1–4 years at the state level in India. Previous studies in India have been cross-sectional [20], examined only infant mortality [21], [22], used data that do not extend into the 2000s [20]–[22] and only focused on public expenditure on health [20]–[22]. This analysis expands the focus to all components of the social sector so as to account for the effects of expenditure on important social determinants of health like education, housing, and water and sanitation. In addition, our models relate expenditure averaged over the five-year period ending in the index year to mortality in that year to account of the cumulative effects of spending on child mortality. Using a five-year average also helps to minimize potential issues of reverse causality, as the effect of health expenditures in one year does not instantly get reflected in the next, especially in Indian states that are themselves the size of many countries [38]. We analyze mortality separately for infants and ages 1–4 years, allowing for a more detailed understanding of the effect of social sector expenditure on these age groups, an important distinction given the differing determinants of mortality at these ages. Furthermore, the data used in this study extend up to 2009, providing an updated estimate of the relationship between social sector expenditure and child mortality in India.

The findings of our analysis are relevant for the 12^th^ Five Year Plan for India (2012–2017), which is the roadmap for India’s budget planning for the coming years. The Planning Commission of India has recommended raising total public expenditure on health to 2.5% of gross domestic product by the end of the 12^th^ Plan, a sizeable increase from the 1.4% that budget estimates indicate was spent in the 2011 fiscal year [39]. It has also suggested emphasis on other aspects of the social sector. However, it remains to be seen how this guidance translates into action, especially with variations among the Indian states in their priorities. In addition, the quality of governance, which too varies among the states, influences the effectiveness of how well the public funds available for the social sector are used for societal benefit.

On a broad level though, the findings in this paper provide evidence that while increases in health expenditure have not been enough to substantially contribute to child mortality reduction in India over the past decade and a half, increases in overall social sector expenditure during this period have contributed to reducing child mortality, indicating that investments in education, housing, urban development and employment are having a beneficial impact on child mortality. Further increases in social sector expenditure, combined with long-term poverty reduction efforts, can play an important role in reducing child mortality in India.

## Supporting Information

Figure S1 **State-wise per-capita health, health-related, education, other, and overall social sector expenditure, 1997–2009.** Per-capita expenditure averaged for the five years ending in the index year. Expenditure is shown in constant 2009 prices (USD).
(TIF)

Figure S2 **State-wise poverty rates (percent of the population below the poverty line), 1997–2009.**
(TIF)

Figure S3 **State-wise death rates in children aged 1–4 years by sex, 1997–2009.**
(TIF)

Figure S4 **State-wise infant mortality rates by sex, 1997–2009.**
(TIF)

Text S1 **Supplemental methods.**
(DOC)

Text S2 **Alternative model specifications.**
(DOC)

File S1 **Supplemental tables.**
(DOC)

File S2 **Random intercepts.**
(DOC)
