# Correction: Social Sector Expenditure and Child Mortality in India: A State-Level Analysis from 1997 to 2009

**DOI:** 10.1371/annotation/e917b690-4e3a-4e90-8545-d4a172617b8f

**Published:** 2013-04-03

**Authors:** Susanna M. Makela, Rakhi Dandona, T. R. Dilip, Lalit Dandona

There were errors in the affiliations in the Author Byline, and in the Author Contributions sections of the article.

The correct affiliations are:

1) Public Health Foundation of India, New Delhi, India

Susanna M. Makela, Rakhi Dandona, T. R. Dilip, Lalit Dandona

2) Institute for Health Metrics and Evaluation, University of Washington, Seattle, Washington, United States of America

Lalit Dandona

The correct Author Contributions are: Conceived and designed the experiments: LD RD. Analyzed the data: SMM. Identified data and contributed to analysis: TRD. Wrote the paper: SMM RD TRD LD. 

