# Supplementary figures and images for: Social Sector Expenditure and Child Mortality in India: A State-Level Analysis from 1997 to 2009
Source: PLoS One. 2013 Feb 7;8(2):e56285. doi: 10.1371/journal.pone.0056285 (PMC3567038; doi:10.1371/journal.pone.0056285)

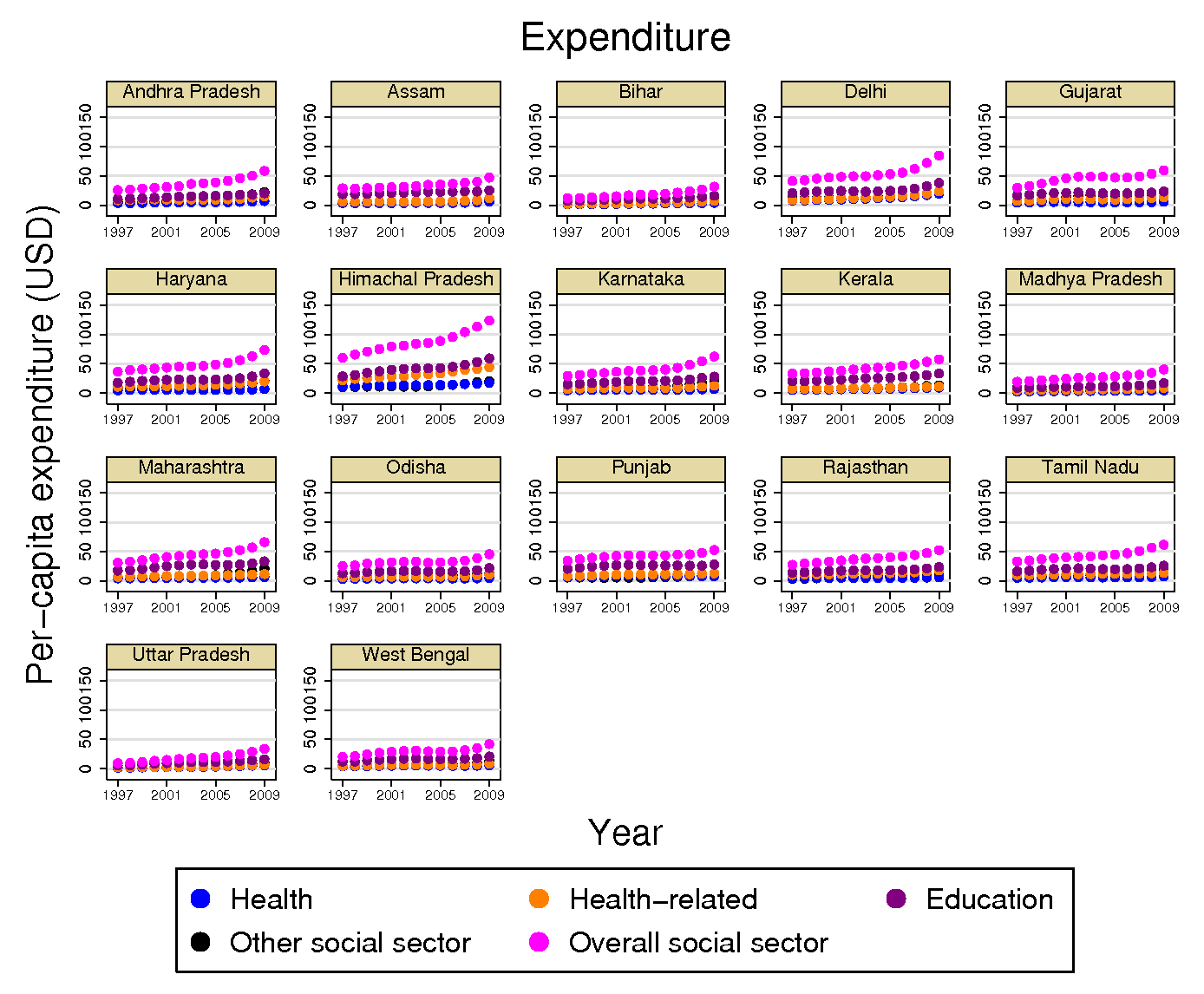

Supplement: Figure S1 — State-wise per-capita health, health-related, education, other, and overall social sector expenditure, 1997–2009. Per-capita expenditure averaged for the five years ending in the index year. Expenditure is shown in constant 2009 prices (USD). (TIF) [file pone.0056285.s001.tif]

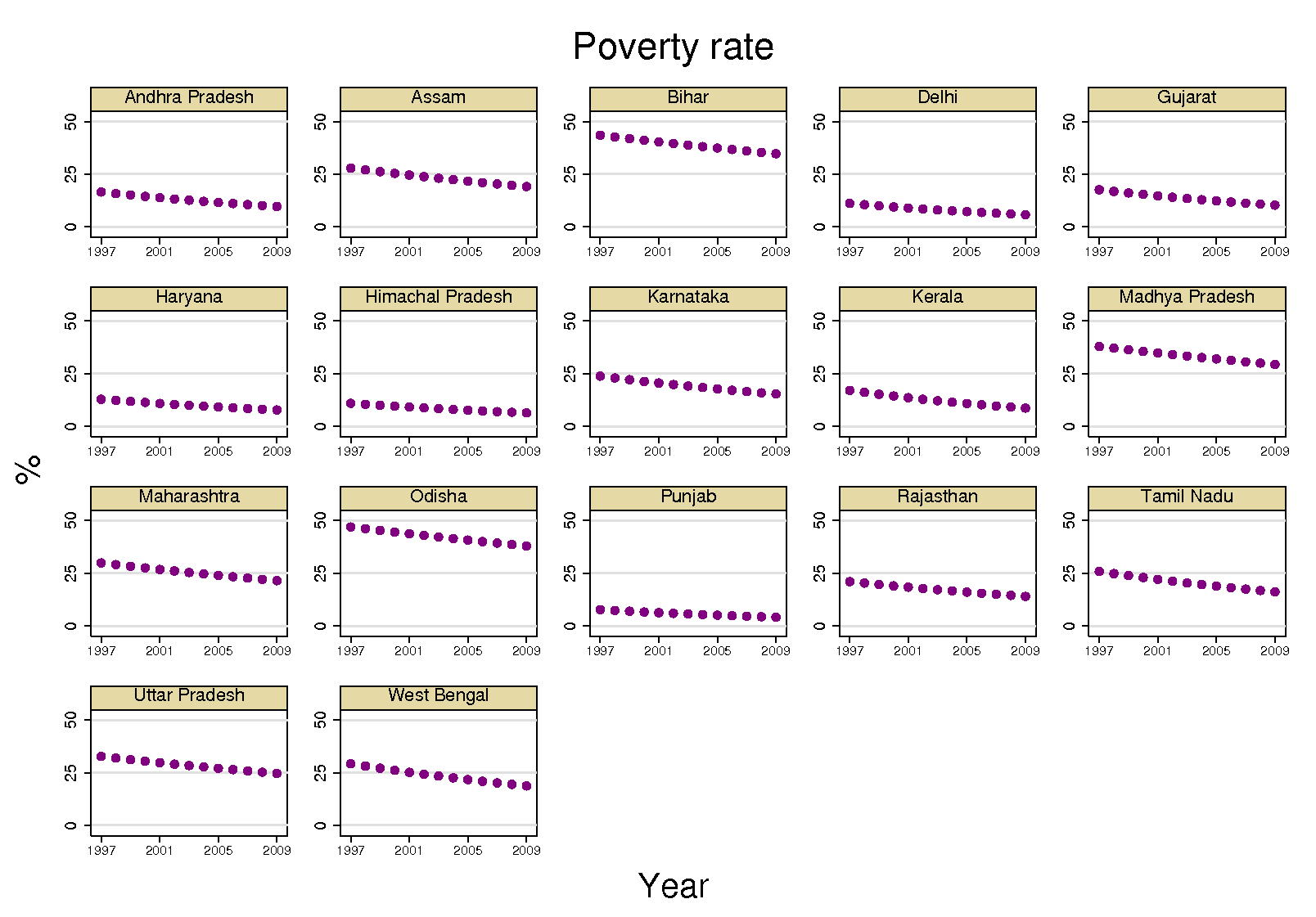

Supplement: Figure S2 — State-wise poverty rates (percent of the population below the poverty line), 1997–2009. (TIF) [file pone.0056285.s002.tif]

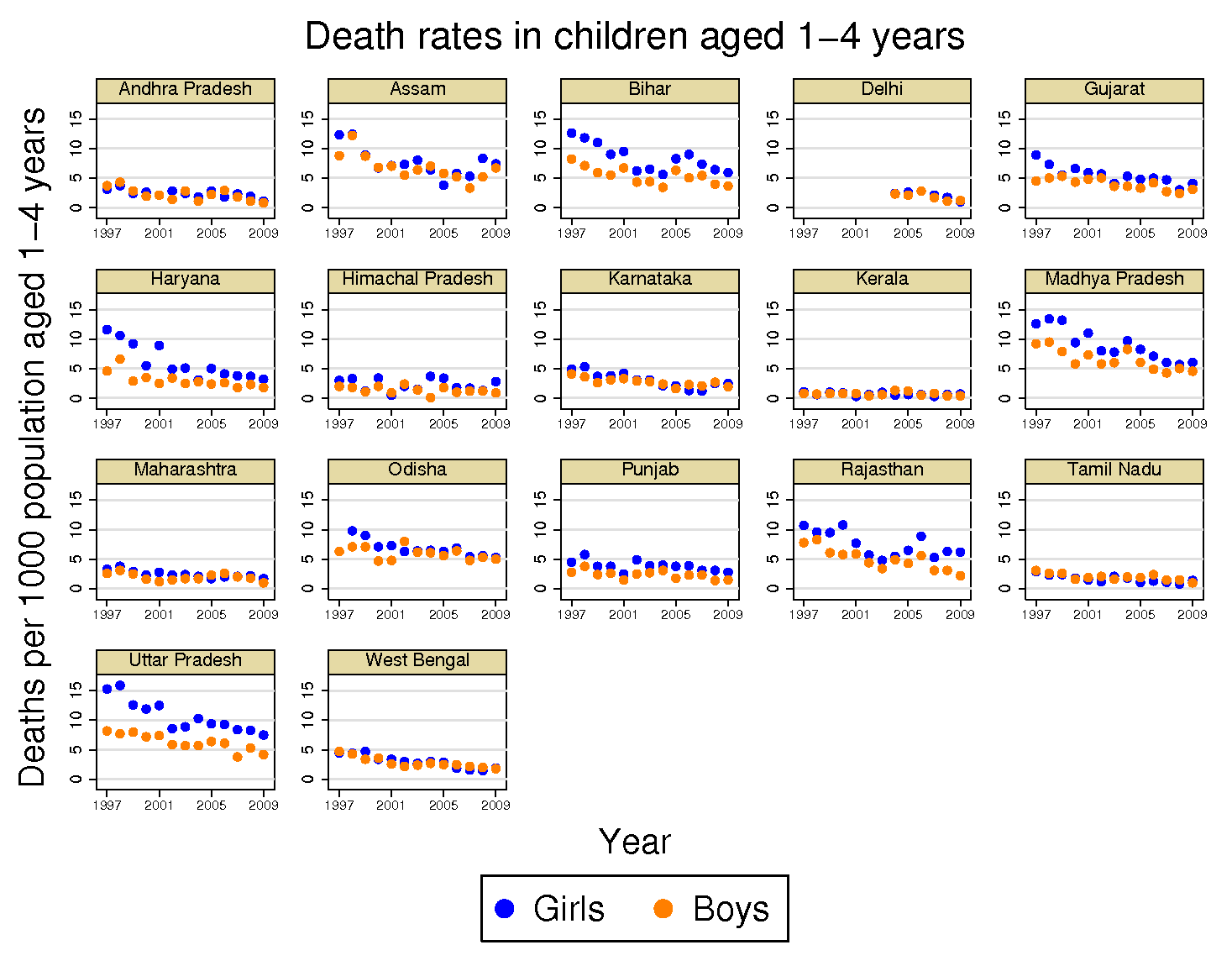

Supplement: Figure S3 — State-wise death rates in children aged 1–4 years by sex, 1997–2009. (TIF) [file pone.0056285.s003.tif]

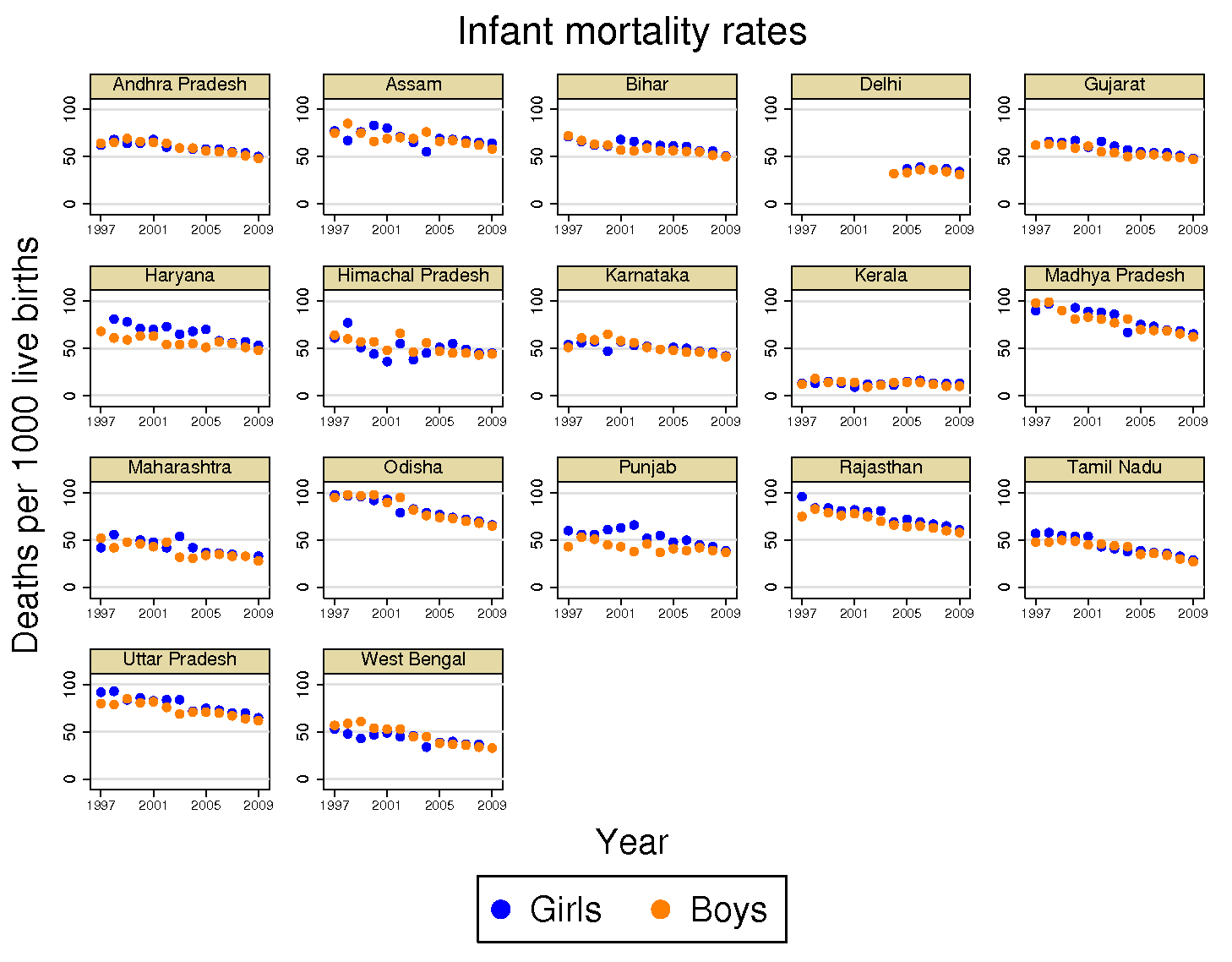

Supplement: Figure S4 — State-wise infant mortality rates by sex, 1997–2009. (TIF) [file pone.0056285.s004.tif]
