# Supplementary material for: Social Sector Expenditure and Child Mortality in India: A State-Level Analysis from 1997 to 2009
Source: PLoS One. 2013 Feb 7;8(2):e56285. doi: 10.1371/journal.pone.0056285 (PMC3567038; doi:10.1371/journal.pone.0056285)
Supplement: Text S1 — Supplemental methods. (DOC) [file pone.0056285.s005.doc]

**Text S1: Supplemental methods**

**Section 1. Combining data for newly-created states**

Data on state-level social sector expenditure are available for Chhattisgarh from 2000 to 2009 and for Jharkhand from 2001 to 2009. We converted these expenditures to constant prices using each state’s respective deflators and added these to the expenditures from the parent state – the states of Madhya Pradesh and Bihar, respectively (also in constant prices).

Mortality data for undivided Bihar and Madhya Pradesh are available until 2003. We combined mortality data for 2004 to 2009 by weighting with the appropriate population denominator: births for infant mortality rates and population aged 1-4 years for death rates at ages 1-4 years. All death rates and population denominators are by sex.

We also added the population totals for Bihar and Jharkhand, and Madhya Pradesh and Chhattisgarh for all years to represent the population of the undivided states, as the original population time series were based on undivided states. Population totals were also added for Uttar Pradesh and Uttarakhand for 1997 to 1999 to get the correct denominator for per-capita expenditure, as expenditure reported prior to 2000 corresponds to undivided Uttar Pradesh.

**Section 2. Population estimates**

**2.1 Total population**

We obtained data on sex-specific population by state for the years 1991, 2001, and 2011 from the Census of India.^1^ We used an exponential growth model to estimate the yearly male and female population from 1997 to 2009. We added male and female populations to calculate total state population, which was used as the denominator for calculating the per-capita state-level social sector expenditure.

**2.2 Population denominators for calculating averted deaths**

To calculate averted deaths, we multiplied the difference between the estimated and counterfactual death rates by the corresponding population in each state. However, annual estimates of population by sex for our age groups of interest were not available at the state level, so we calculated these based on existing data. Below, we summarize the data and methods used to calculate these population numbers.

**2.2.1 Births**

The denominator of the infant mortality rate is births per year. For each state-year, the number of births by sex was calculated using

$$births_{it}=cbr_{it}*P_{it}*SRB_{it}$$

where *i* indexes states, *t* indexes years, *cbr_it_* is the crude birth rate from the Sample Registration System (SRS), *P_it_* is the total sex-specific population, and *SRB_it_* is the sex ratio at birth in the state from the SRS.^2^

We smoothed the crude birth rate using a three-year moving average to minimize the effect of noise in the raw data.

Because the sex ratio is reported as the number of girls per 1000 boys, we set  *SRB_it_* equal to the reported sex ratio divided by (the reported sex ratio + 1000) when calculating births of girls and to one minus that quantity when calculating births of boys. For example, if the reported sex ratio were 950, we would use  *SRB_it_* = (950/(950+1000)) = .49 for girls and  *SRB_it_* = 1–.49 = .51 for boys.

The SRB is reported by the SRS for children under age 6 years in three-year intervals from 1998-2000 to 2007-2009. We assigned the reported values to the midpoint of each three-year interval to obtain a time series of sex ratios from 1999 to 2008. We extended the time series to 1997 and 2009 by assigning the 1999 sex ratio to 1997 and 1998 and the 2008 sex ratio to 2009. This type of simple imputation was used as we observed only marginal variations in the raw SRB data, with 63% of successive years exhibiting a change of less than 10 girls per 1000 boys and 86% a change of less than 20 girls per 1000 boys.

The SRS only reports the SRB for Delhi from 2003 to 2008. We used data from the National Family Health Survey (NFHS) round 2 (1998-1999)^3^ and round 3 (2005-2006)^4^ to estimate the SRB from 1997 to 2002. For each year from 1997 to 2002, we calculated the total number of boys and girls born in the five-year interval centered at the index year and divided to obtain the five-year average SRB.

As with the crude birth ratio, we smoothed the sex ratios with a three-year moving average.

**2.2.2 Population aged 1-4 years**

The population aged 1-4 is the denominator for the death rate at ages 1-4 years. This quantity was calculated by multiplying the population aged 0-4 by the percentage of that population aged 1-4 years. We used 1991 and 2001 census data along with an exponential growth model to calculate yearly percentages of the population aged 1-4 years out of the age 0-4 population. We then regressed these percentages on the crude birth rate separately within each state to extrapolate the percentages for 2002 to 2009. We used the crude birth rate because it is the major determinant – lesser determinants being child mortality and migration rates – of the relative composition of children aged less than one year and children aged 1-4 years in the 0-4 population.

**2.2.3 Population aged 0-4 years**

The population aged 0-4 years was calculated by multiplying the total sex-specific population by the sex-specific percentage of the population aged 0-4 years:

$$pop0-4_{it}=P_{it}*(\%pop0-4_{it})$$

We calculated the sex-specific total population *P_it_* as described previously. Data on the percent of the population aged 0-4 years by sex was obtained from the SRS. As these data contained implausible fluctuations, we regressed the percent of the population aged 0-4 by sex on year in order to obtain more realistic estimates of this percentage in each state from 1997 to 2009.

We did not have data on the percent of the population aged 0-4 years for Delhi from 1991 to 2003. To fill this gap, we used census data from 1991 and 2001 and an exponential growth model to estimate the percentage of the population aged 0-4 years from 1991 to 2003.

**Section 3. Autocorrelation**

As time series cross-sectional data often suffer from autocorrelation, we tested our model specification with overall social sector expenditure for serial autocorrelation using the Wooldridge test^6^ with the Stata command xtserial. Evidence for autocorrelation is seen only for infant girls, for which group there was no significant relationship between overall social sector expenditure and mortality rates.

**Table A. Autocorrelation by sex and age in model with overall social sector expenditure**

| **Sex** | **Age** | **p-value** |
| --- | --- | --- |
| Boys | infants | 0.1456 |
| Boys | 1-4 | 0.7369 |
| Girls | infants | 0.0033 |
| Girls | 1-4 | 0.7791 |

**Section 4. Simulation-based uncertainty intervals for averted deaths**

$$\ln(dr_{it})=\beta_{0}+\beta_{1}\ln(\exp_{it})+\beta_{2}(pov_{it})+\eta_{i}+\eta_{t}+\varepsilon_{it}$$

1. 1000 samples of the fixed-effects parameters (*β_0_, β_1_, β_2_* in the model above) were drawn from a multivariate normal distribution using the point estimates and variance-covariance matrix.
2. 1000 samples of the random effects *η_i_* and *η_t_* were each drawn from a normal distribution using the BLUP (best linear unbiased prediction) as the mean and the estimated standard error of the BLUP as the standard deviation.
3. Predictions of the observed and counterfactual death rates were calculated based on the above parameters to generate 1000 pairs of observed and counterfactual death rates.
4. The difference between the predicted observed and counterfactual death rates was multiplied by the appropriate population denominator to calculate 1000 simulations of the number of averted deaths.
5. The 2.5% and 97.5% percentiles of the 1000 simulated deaths averted were used as the uncertainty intervals.

Table B below lists averted deaths by state in 2009 attributable to increases in social sector expenditure and decreases in poverty since 1997.

**Table B. Averted deaths in 2009 by state.** Observed deaths in 1997 and 2009, as well as deaths by state in 2009 attributable to increases in social sector expenditure and decreases in poverty since 1997.

|  |  |  | **Observed Deaths** | | **Averted deaths (95% UI) in 2009 attributed to improvements in** | |
| --- | --- | --- | --- | --- | --- | --- |
| **Sex** | **Age** | **State** | **1997** | **2009** | **Overall social sector expenditure** | **Poverty** |
| boys | infants | Andhra Pradesh | 56,281 | 39,451 | 0 (0 – 4,502) | 14,155 (9,014 – 21,584) |
| boys | infants | Assam | 28,799 | 20,620 | 0 (0 – 1,205) | 9,909 (5,726 – 15,779) |
| boys | infants | Bihar | 115,117 | 88,977 | 0 (0 – 10,420) | 42,833 (23,827 – 74,326) |
| boys | infants | Delhi | 5,341 | 4,913 | 0 (0 – 453) | 1,331 (887 – 1,967) |
| boys | infants | Gujarat | 40,899 | 31,333 | 0 (0 – 2,821) | 12,124 (7,717 – 19,108) |
| boys | infants | Haryana | 18,600 | 14,876 | 0 (0 – 1,334) | 3787 (2441 – 5,816) |
| boys | infants | Himachal Pradesh | 3,829 | 2,728 | 0 (0 – 244) | 616 (382 – 967) |
| boys | infants | Karnataka | 35,165 | 25,017 | 0 (0 – 2,437) | 11,577 (7,155 – 18,192) |
| boys | infants | Kerala | 4,339 | 2,544 | 0 (0 – 188) | 1,163 (729 – 1,747) |
| boys | infants | Madhya Pradesh | 114,298 | 83,847 | 0 (0 – 7,337) | 38,665 (21,649 – 64,058) |
| boys | infants | Maharashtra | 48,378 | 31,506 | 0 (0 – 2,933) | 14,454 (8,399 – 24,960) |
| boys | infants | Odisha | 46,642 | 29,232 | 0 (0 – 1,911) | 14,554 (7,582 – 27,863) |
| boys | infants | Punjab | 13,106 | 9,594 | 0 (0 – 585) | 1,676 (1,085 – 2,518) |
| boys | infants | Rajasthan | 69,620 | 55,968 | 0 (0 – 4,956) | 20,706 (12,654 – 31,748) |
| boys | infants | Tamil Nadu | 29,372 | 18,771 | 0 (0 – 1,518) | 10,155 (6,095 – 16,393) |
| boys | infants | Uttar Pradesh | 231,599 | 176,968 | 0 (0 – 31,604) | 77,101 (47,458 – 130,610) |
| boys | infants | West Bengal | 48,333 | 28,095 | 0 (0 – 2,506) | 16,841 (9,861 – 26,935) |
| girls | infants | Andhra Pradesh | 54,049 | 37,059 | 2,414 (0 – 8,099) | 8,794 (4,903 – 14,198) |
| girls | infants | Assam | 27,573 | 20,106 | 774 (0 – 2,425) | 6,299 (3,111 – 10,627) |
| girls | infants | Bihar | 107,849 | 88,553 | 6,853 (0 – 20,144) | 27,787 (12,478 – 52,358) |
| girls | infants | Delhi | 4,736 | 4,611 | 265 (0 – 867) | 835 (490 – 1,271) |
| girls | infants | Gujarat | 37,109 | 31,338 | 1,742 (0 – 5,762) | 7,993 (4,269 – 12,978) |
| girls | infants | Haryana | 17,389 | 14,909 | 830 (0 – 2,684) | 2,543 (1,417 – 4,007) |
| girls | infants | Himachal Pradesh | 3,337 | 2,482 | 142 (0 – 453) | 377 (209 – 625) |
| girls | infants | Karnataka | 32,009 | 23,953 | 1,452 (0 – 4,632) | 7,241 (3,639 – 12,207) |
| girls | infants | Kerala | 3,998 | 2,600 | 115 (0 – 394) | 777 (424 – 1,221) |
| girls | infants | Madhya Pradesh | 104,799 | 83,671 | 4,907 (0 – 14,216) | 25,210 (11,561 – 46,205) |
| girls | infants | Maharashtra | 48,149 | 32,271 | 1,959 (0 – 5,997) | 9,675 (4,614 – 17,668) |
| girls | infants | Odisha | 42,114 | 28,682 | 1,337 (0 – 3,487) | 9,291 (3,784 – 19,307) |
| girls | infants | Punjab | 13,054 | 10,163 | 344 (0 – 1,207) | 1,202 (714 – 1,758) |
| girls | infants | Rajasthan | 65,743 | 54,723 | 2,826 (0 – 9,219) | 13,371 (7,121 – 21,736) |
| girls | infants | Tamil Nadu | 28,298 | 19,452 | 964 (0 – 3,017) | 6,813 (3,425 – 11,740) |
| girls | infants | Uttar Pradesh | 218,671 | 168,285 | 17,794 (0 – 56,521) | 48,045 (23,187 – 83,033) |
| girls | infants | West Bengal | 41,221 | 25,693 | 1,491 (0 – 4,932) | 9,908 (4,942 – 17,105) |
| boys | 1 to 4 | Andhra Pradesh | 8,860 | 3,710 | 2,697 (1,099 – 5,342) | 705 (76 – 1,424) |
| boys | 1 to 4 | Assam | 10,249 | 6,372 | 2,466 (1,185 – 4,416) | 1,590 (146 – 3,698) |
| boys | 1 to 4 | Bihar | 49,590 | 24,224 | 21,959 (10,658 – 38,653) | 6,055 (414 – 17,525) |
| boys | 1 to 4 | Delhi | 1,477 | 914 | 569 (256 – 1,038) | 133 (14 – 252) |
| boys | 1 to 4 | Gujarat | 12,383 | 7,410 | 4,427 (1,894 – 8,287) | 1,512 (135 – 3,188) |
| boys | 1 to 4 | Haryana | 3,990 | 2,169 | 1,297 (561 – 2,580) | 298 (35 – 611) |
| boys | 1 to 4 | Himachal Pradesh | 432 | 210 | 130 (62 – 233) | 26 (3 – 55) |
| boys | 1 to 4 | Karnataka | 8,258 | 3,885 | 2,581 (1,185 – 4,718) | 936 (91 – 2,061) |
| boys | 1 to 4 | Kerala | 1,119 | 552 | 250 (105 – 492) | 132 (13 – 259) |
| boys | 1 to 4 | Madhya Pradesh | 36,753 | 18,920 | 12,068 (6,169 – 20,623) | 4,545 (340 – 12,213) |
| boys | 1 to 4 | Maharashtra | 12,035 | 5,655 | 3,763 (1,884 – 6,249) | 1,352 (117 – 3,633) |
| boys | 1 to 4 | Odisha | 13,366 | 6,956 | 3,364 (1,958 – 5,359) | 1,794 (111 – 6,159) |
| boys | 1 to 4 | Punjab | 2,936 | 1,901 | 634 (235 – 1,373) | 182 (24 – 318) |
| boys | 1 to 4 | Rajasthan | 18,004 | 11,350 | 6,202 (2,635 – 11,788) | 2,221 (229 – 4,839) |
| boys | 1 to 4 | Tamil Nadu | 6,497 | 3,133 | 1,630 (748 – 2,851) | 872 (74 – 2,053) |
| boys | 1 to 4 | Uttar Pradesh | 97,340 | 37,471 | 51,982 (20,652 – 105,611) | 8,540 (827 – 20,219) |
| boys | 1 to 4 | West Bengal | 13,872 | 6,017 | 3,787 (1,639 – 7,153) | 1,839 (168 – 4,362) |
| girls | 1 to 4 | Andhra Pradesh | 9,779 | 4,304 | 1,714 (274 – 4,745) | 1,803 (757 – 3,468) |
| girls | 1 to 4 | Assam | 12,189 | 6,954 | 1,546 (290 – 3,405) | 3,929 (1,450 – 8,492) |
| girls | 1 to 4 | Bihar | 72,263 | 37,175 | 18,054 (3,453 – 37,836) | 21,039 (6,051 – 58,934) |
| girls | 1 to 4 | Delhi | 1,550 | 1,008 | 349 (58 – 848) | 317 (145 – 548) |
| girls | 1 to 4 | Gujarat | 15,189 | 9,040 | 3,010 (485 – 7,710) | 4,091 (1,796 – 7,799) |
| girls | 1 to 4 | Haryana | 6,375 | 3,569 | 1,189 (198 – 2,801) | 1,054 (448 – 2,054) |
| girls | 1 to 4 | Himachal Pradesh | 628 | 347 | 120 (24 – 271) | 91 (34 – 187) |
| girls | 1 to 4 | Karnataka | 8,651 | 4,056 | 1,488 (261 – 3,335) | 2,205 (791 – 4,735) |
| girls | 1 to 4 | Kerala | 1,019 | 492 | 127 (19 – 348) | 264 (120 – 495) |
| girls | 1 to 4 | Madhya Pradesh | 50,174 | 26,380 | 9,325 (1,873 – 19,785) | 14,286 (4,171 – 34,842) |
| girls | 1 to 4 | Maharashtra | 14,148 | 6,395 | 2,350 (489 – 4,848) | 3,445 (1,092 – 8,210) |
| girls | 1 to 4 | Odisha | 15,348 | 7,499 | 2,053 (547 – 3,838) | 4,393 (929 – 13,444) |
| girls | 1 to 4 | Punjab | 4,047 | 2,633 | 508 (70 – 1,536) | 531 (260 – 903) |
| girls | 1 to 4 | Rajasthan | 25,870 | 16,461 | 5,047 (795 – 12,697) | 7,115 (2,874 – 13,519) |
| girls | 1 to 4 | Tamil Nadu | 5,455 | 2,457 | 720 (135 – 1,663) | 1,567 (579 – 3,311) |
| girls | 1 to 4 | Uttar Pradesh | 139,971 | 62,855 | 44,338 (7,096 – 113,762) | 32,113 (10,533 – 68,837) |
| girls | 1 to 4 | West Bengal | 14,557 | 6,013 | 2,100 (359 – 5,032) | 42,64 (1,514 – 8,987) |
