# Supplementary material for: Social Sector Expenditure and Child Mortality in India: A State-Level Analysis from 1997 to 2009
Source: PLoS One. 2013 Feb 7;8(2):e56285. doi: 10.1371/journal.pone.0056285 (PMC3567038; doi:10.1371/journal.pone.0056285)
Supplement: Text S2 — Alternative model specifications. (DOC) [file pone.0056285.s006.doc]

**Text S2: Alternative model specifications**

**Section 1. Poverty and illiteracy**

In addition to controlling for poverty rates in our main model, we tested the effect of including a measure of illiteracy both along with and insteaad of poverty. Illiteracy rates of women aged 15-35 by state were obtained from the Planning Commission of India for the years 1973-74, 1977-78, 1983, 1987-88, 1993-94, 1999-2000, and 2004-05.^5^ We predicted illiteracy rates for the missing years with a method analogous to that used to predict poverty rates. We regressed log illiteracy rates on year in each state and then predicted annual illiteracy rates for the period 1997-2009.

We refer to the model with log per-capita overall social sector expenditure and poverty rates, the model presented in the main body of the paper, as Model 1. Model 2 is the same as Model 1 but replaces poverty rates with illiteracy rates, and Model 3 includes illiteracy rates in addition to poverty rates. All three models include random intercepts by state and year. Expenditure is measured in log per-capita terms and is the average of the five years ending in the index year

As seen in table A, the coefficient for log per-capita overall social sector expenditure is not significant in any of the three models for infants. In ages 1-4, this coefficient is significant for both sexes in Models 1 and 3 and for boys in Model 2. The magnitude of this coefficient is similar for ages 1-4 in Models 1 and 2 (-0.68 and -0.51 for boys, -0.41 and -0.36 for girls, respectively), but somewhat smaller in Model 2 (-0.41 for boys, -0.20 for girls). Confidence intervals for the coefficients for overall social sector expenditure overlap in all three models, and measures of model fit (median absolute error and median relative error; data not shown) between the three models are very similar.

We chose to retain poverty instead of illiteracy rates because the coefficients between the three models above are largely consistent. In addition, the main aim of this analysis was to identify the effect of state-level overall social sector expenditure in the context of a broad analysis of social determinants of health, and poverty is a social determinant that itself influences many other factors that impact health status.

**Table A. Comparison of results from regression models of mortality and death rates on per-capita state overall social sector expenditure while controlling for:**

1. **poverty (Model 1)**
2. **illiteracy (Model 2)**
3. poverty and illiteracy (Model 3)

| **Age-sex group** | **Model term** | **Poverty (Model 1)** | **Illiteracy (Model 2)** | **Poverty and illiteracy (Model 3)** |
| --- | --- | --- | --- | --- |
| **Infants, boys** |  |  |  |  |
|  | Overall social sector expenditure | 0.00 (-0.12 to 0.12) | -0.09 (-0.21 to 0.04) | 0.08 (-0.05 to 0.21) |
|  | Poverty | 0.05 (0.03 to 0.06) ^a^ | - | 0.03 (0.02 to 0.04) ^a^ |
|  | Illiteracy | - | 0.02 (0.02 to 0.03) ^a^ | 0.01 (0.01 to 0.02) ^a^ |
|  | Intercept | 3.00 (1.88 to 4.12) ^a^ | 3.77 (2.67 to 4.88) ^a^ | 2.21 (1.00 to 3.42) ^a^ |
|  | σ_t_ (year) | 0.02 (0.01 to 0.05) ^a^ | 0.03 (0.01 to 0.07) ^a^ | 0.03 (0.01 to 0.05) ^a^ |
|  | σ_i_ (state) | 0.49 (0.34 to 0.70) ^a^ | 0.32 (0.22 to 0.45) ^a^ | 0.40 (0.27 to 0.59) ^a^ |
| **Infants, girls** |  |  |  |  |
|  | Overall social sector expenditure | -0.08 (-0.22 to 0.06) | 0.06 (-0.07 to 0.19) | 0.11 (-0.03 to 0.26) |
|  | Poverty | 0.03 (0.02 to 0.04) ^a^ | - | 0.01 (-0.00 to 0.02) |
|  | Illiteracy | - | 0.03 (0.02 to 0.04) ^a^ | 0.03 (0.02 to 0.03) ^a^ |
|  | Intercept | 3.92 (2.63 to 5.21) ^a^ | 2.56 (1.39 to 3.72) ^a^ | 2.03 (0.72 to 3.35) ^a^ |
|  | σ_t_ (year) | 0.02 (0.01 to 0.06) ^a^ | 0.02 (0.01 to 0.06) ^a^ | 0.03 (0.01 to 0.06) ^a^ |
|  | σ_i_ (state) | 0.44 (0.31 to 0.64) ^a^ | 0.33 (0.23 to 0.47) ^a^ | 0.33 (0.23 to 0.48) ^a^ |
| **1-4 years, boys** |  |  |  |  |
|  | Overall social sector expenditure | -0.68 (-1.00 to -0.35) ^a^ | -0.51 (-0.82 to -0.20) ^a^ | -0.41 (-0.76 to -0.05) ^a^ |
|  | Poverty | 0.03 (0.00 to 0.05) ^a^ | - | 0.01 (-0.01 to 0.03) |
|  | Illiteracy | - | 0.03 (0.01 to 0.04) ^a^ | 0.02 (0.01 to 0.04) ^a^ |
|  | Intercept | 5.59 (2.77 to 8.42) ^a^ | 3.99 (1.30 to 6.68) ^a^ | 3.07 (-0.04 to 6.18) |
|  | σ_t_ (year) | 0.04 (0.01 to 0.28) ^a^ | 0.05 (0.01 to 0.21) ^a^ | 0.05 (0.02 to 0.19) ^a^ |
|  | σ_i_ (state) | 0.46 (0.32 to 0.67) ^a^ | 0.42 (0.29 to 0.61) ^a^ | 0.41 (0.28 to 0.61) ^a^ |
| **1-4 years, girls** |  |  |  |  |
|  | Overall social sector expenditure | -0.41 (-0.75 to -0.08) ^a^ | -0.36 (-0.66 to -0.05) ^a^ | -0.20 (-0.54 to 0.15) |
|  | Poverty | 0.05 (0.02 to 0.08) ^a^ | - | 0.02 (-0.00 to 0.05) |
|  | Illiteracy | - | 0.04 (0.02 to 0.05) ^a^ | 0.03 (0.01 to 0.05) ^a^ |
|  | Intercept | 3.38 (0.39 to 6.36) ^a^ | 2.70 (0.01 to 5.39) ^a^ | 1.22 (-1.90 to 4.33) |
|  | σ_t_ (year) | 0.04 (0.01 to 0.19) ^a^ | 0.04 (0.01 to 0.19) ^a^ | 0.04 (0.01 to 0.21) ^a^ |
|  | σ_i_ (state) | 0.68 (0.46 to 1.00) | 0.51 (0.35 to 0.74) ^a^ | 0.54 (0.37 to 0.80)^a^ |

**for infants and age 1-4 years by sex for the years 1997-2009.**

95% confidence intervals given in parentheses.

^a^Statistically significant coefficient (5% level)

**Section 2. Nutrition and water/sanitation expenditure**

In addition to exploring health and health-related expenditure, we also investigated the effect of expenditure on nutrition and water/sanitation by itself (nutrition and water/sanitation is the difference between health and health-related expenditure). Table B below shows results for the main model with overall social sector expenditure (Model 1) and the model with nutrition and water/sanitation expenditure (Model 2). Expenditure on nutrition and water/sanitation is not significantly associated with mortality in infants or at ages 1-4 years.

All expenditure is measured in log per-capita terms and is the average of the five years ending in the index year, and both models adjust for poverty rates and include random intercepts by state and year.

**Table B. Comparison of results from regression models of mortality and death rates on the following measures of expenditure:**

1. **overall social sector (Model 1)**
2. **nutrition & water/sanitation (Model 2)**

**for infants and ages 1-4 years by sex for the years 1997-2009.**

| **Age-sex group** | **Model term** | **Overall (Model 1)** | **Nutrition & water/sanitation (Model 2)** |
| --- | --- | --- | --- |
| **Infants, boys** |  |  |  |
|  | Expenditure | 0.00 (-0.12 to 0.12) | 0.04 (-0.03 to 0.11) |
|  | Poverty | 0.05 (0.03 to 0.06) ^a^ | 0.05 (0.04 to 0.06) ^a^ |
|  | Intercept | 3.00 (1.88 to 4.12) ^a^ | 2.77 (2.26 to 3.29) ^a^ |
|  | σ_t_ (year) | 0.02 (0.01 to 0.05) ^a^ | 0.03 (0.01 to 0.06) ^a^ |
|  | σ_i_ (state) | 0.49 (0.34 to 0.70) ^a^ | 0.48 (0.36 to 0.70) ^a^ |
| **Infants, girls** |  |  |  |
|  | Expenditure | -0.08 (-0.22 to 0.06) | -0.05 (-0.13 to 0.03) |
|  | Poverty | 0.03 (0.02 to 0.04) ^a^ | 0.03 (0.02 to 0.04) ^a^ |
|  | Intercept | 3.92 (2.63 to 5.21) ^a^ | 3.57 (2.98 to 4.16) ^a^ |
|  | σ_t_ (year) | 0.02 (0.01 to 0.06) ^a^ | 0.02 (0.01 to 0.06) ^a^ |
|  | σ_i_ (state) | 0.44 (0.31 to 0.64) ^a^ | 0.46 (0.32 to 0.66) ^a^ |
| **1-4 years, boys** |  |  |  |
|  | Expenditure | -0.68 (-1.00 to -0.35) ^a^ | -0.13 (-0.33 to 0.07) |
|  | Poverty | 0.03 (0.00 to 0.05) ^a^ | 0.05 (0.03 to 0.07) ^a^ |
|  | Intercept | 5.59 (2.77 to 8.42) ^a^ | 0.77 (-0.56 to 2.09) |
|  | σ_t_ (year) | 0.04 (0.01 to 0.28) ^a^ | 0.10 (0.04 to 0.22) ^a^ |
|  | σ_i_ (state) | 0.46 (0.32 to 0.67) ^a^ | 0.53 (0.36 to 0.77) ^a^ |
| **1-4 years, girls** |  |  |  |
|  | Expenditure | -0.41 (-0.75 to -0.08) ^a^ | -0.08 (-0.28 to 0.12) |
|  | Poverty | 0.05 (0.02 to 0.08) ^a^ | 0.07 (0.05 to 0.10) ^a^ |
|  | Intercept | 3.38 (0.39 to 6.36) ^a^ | 0.21 (-1.18 to 1.60) |
|  | σ_t_ (year) | 0.04 (0.01 to 0.19) ^a^ | 0.05 (0.01 to 0.19) ^a^ |
|  | σ_i_ (state) | 0.68 (0.46 to 1.00) | 0.79 (0.54 to 1.15) |

95% confidence intervals given in parentheses.

^a^Statistically significant coefficient (5% level)

**Section 3. Other model specifications**

In addition to the inclusion of alternative covariates and measures of expenditure presented above, we also explored other possible model specifications. First, we considered a first-differences model, which uses the difference in mortality/death rates between years t+1 and t as the dependent variable and the differences in overall social sector expenditure and poverty rates between years t+1 and t as covariates. This model does not include random intercepts by state, as they cancel out in the differencing. The first differences model is useful when there exist unmeasured covariates that do not change over time (e.g. cultural, geographic, climate, historical, and other factors particular to each state) and when the observed covariates are observed with errors that persist over time [1], which is likely to be the case with the poverty variable (although we note that a test for autocorrelation was significant only for infant girls, as shown in table A of supplemental text S1). We also considered a model in which we use indicator variables for each state instead of random intercepts by state. We do not show the coefficients for the indicator variables for the sake of clarity.

Finally, we pooled the sex-specific mortality data in each state-year and added an indicator for female sex and an expenditure-female sex interaction:

$$\ln(dr_{it})=\beta_{0}+\beta_{1}\ln(\text{exp}_{it})+\beta_{2}(female)+\beta_{3}(female*\ln(\exp_{it}))+\beta_{4}(pov_{it})+\eta_{i}+\eta_{t}+\varepsilon_{it}$$

All models used overall social sector expenditure as the expenditure measure.

As shown in table C, the results from the main model used in the main text are largely consistent with the results from the first differences model and the model using state indicator variables, particularly for boys aged 1-4 years, the only age group for which overall social sector expenditure was significant. The results from the model in table D are also consistent with this result. The coefficient *β_1_* on overall social sector expenditure in the equation above can be interpreted as the association between expenditure and death rates for boys, which is significant for the 1-4 year age group. The sum *β_2_* + *β_3_* represents the association between expenditure and death rates in girls, and while it is not significant for girls aged 1-4 years, the magnitude of the coefficient is very similar to that in the models in table C.

**Table C. Comparison of results from regression models of mortality and death rates on overall social sector expenditure for infants and ages 1-4 years by sex for the years 1997-2009 using:**

1. **main model (Model 1)**
2. **first differences (Model 2)**
3. **state indicator variables (Model 3)**

| **Age-sex group** | **Model term** | **Main model (Model 1)** | **First differences (Model 2)** | **State indicator variables (Model 3)** |
| --- | --- | --- | --- | --- |
| **Infants, boys** |  |  |  |  |
|  | Overall social sector expenditure | 0.00 (-0.12 to 0.12) | -0.20 (-0.55 to 0.15) | 0.05 (-0.09 to 0.17) |
|  | Poverty | 0.05 (0.03 to 0.06) ^a^ | 0.00 (-0.01 to 0.01) | 0.05 (0.04 to 0.06) ^a^ |
|  | Intercept | 3.00 (1.88 to 4.12) ^a^ | -0.02 (-0.07 to 0.04) | 3.10 (2.03 to 4.18) ^a^ |
|  | σ_t_ (year) | 0.02 (0.01 to 0.05) ^a^ | 0.02 (0.00 to 0.06) ^a^ | 0.02 (0.01 to 0.05) ^a^ |
| **Infants, girls** |  |  |  |  |
|  | Overall social sector expenditure | -0.08 (-0.22 to 0.06) | 0.05 (-0.37 to 0.47) | -0.03 (-0.18 to 0.13) |
|  | Poverty | 0.03 (0.02 to 0.04) ^a^ | 0.01 (-0.09 to 0.10) | 0.04 (0.02 to 0.05) ^a^ |
|  | Intercept | 3.92 (2.63 to 5.21) ^a^ | -0.03 (-0.10 to 0.04) | 3.83 (2.53 to 5.12) ^a^ |
|  | σ_t_ (year) | 0.02 (0.01 to 0.06) ^a^ | 0.03 (0.02 to 0.07) ^a^ | 0.02 (0.01 to 0.06) ^a^ |
| **1-4 years, boys** |  |  |  |  |
|  | Overall social sector expenditure | -0.68 (-1.00 to -0.35) ^a^ | -0.65 (-2.04 to 0.74) | -0.57 (-1.02 to -0.11) ^a^ |
|  | Poverty | 0.03 (0.00 to 0.05) ^a^ | -0.03 (-0.37 to 0.31) | 0.04 (-0.00 to 0.08) |
|  | Intercept | 5.59 (2.77 to 8.42) ^a^ | -0.05 (-0.29 to 0.20) | 4.43 (0.60 to 8.27) ^a^ |
|  | σ_t_ (year) | 0.04 (0.01 to 0.28) ^a^ | 0.03 (0.00 to 123.27) | 0.05 (0.01 to 0.24) ^a^ |
| **1-4 years, girls** |  |  |  |  |
|  | Overall social sector expenditure | -0.41 (-0.75 to -0.08) ^a^ | -0.41 (-1.57 to 0.74) | -0.15 (-0.55 to 0.24) |
|  | Poverty | 0.05 (0.02 to 0.08) ^a^ | 0.07 (-0.21 to 0.36) | 0.08 (0.04 to 0.11) ^a^ |
|  | Intercept | 3.38 (0.39 to 6.36) ^a^ | 0.01 (-0.19 to 0.22) | 0.94 (-2.40 to 4.28) |
|  | σ_t_ (year) | 0.04 (0.01 to 0.19) ^a^ | 0.00 (0.00 to 0.00) ^a^ | 0.33 (0.00 to 0.26) ^a^ |

95% confidence intervals given in parentheses.

State fixed effects in Model 2 not shown for clarity.

^a^Statistically significant coefficient (5% level)

**Table D. Results from a regression model of mortality and death rates on overall social sector expenditure for infants and ages 1-4 years for the years 1997-2009 with a sex-expenditure interaction.**

| **Age group** | **Model term** | **Coefficient (95% CI)** |
| --- | --- | --- |
| **Infants** |  |  |
|  | Overall social sector expenditure | 0.07 (-0.10 to 0.12) |
|  | Overall social sector expenditure x female | -0.02 (-0.06 to 0.02) |
|  | Female | 0.11 (-0.21 to 0.42) |
|  | Poverty | 0.04 (0.03 to 0.05) ^a^ |
|  | Intercept | 3.11 (2.12 to 4.10) ^a^ |
|  | σ_t_ (year) | 0.03 (0.01 to 0.05) ^a^ |
|  | σ_i_ (state) | 0.47 (0.32 to 0.67) ^a^ |
| **1-4 years** |  |  |
|  | Overall social sector expenditure | -0.50 (-0.79 to -0.21) ^a^ |
|  | Overall social sector expenditure x female | 0.01 (-0.12 to 0.14) |
|  | Female | -0.35 (-1.34 to 0.64) |
|  | Poverty | 0.40 (0.02 to 0.06) ^a^ |
|  | Intercept | 4.22 (1.68 to 6.78) ^a^ |
|  | σ_t_ (year) | 0.05 (0.02 to 0.14) ^a^ |
|  | σ_i_ (state) | 0.56 (0.38 to 0.81) ^a^ |

95% confidence intervals given in parentheses.

^a^Statistically significant coefficient (5% level)

**References**

1. Liker JK, Augustyniak S, Duncan GJ (1985) Panel data and models of change: A comparison of first differences and conventional two-wave models. Social Science Research 14: 80-101.
