# Supplementary material for: Social Sector Expenditure and Child Mortality in India: A State-Level Analysis from 1997 to 2009
Source: PLoS One. 2013 Feb 7;8(2):e56285. doi: 10.1371/journal.pone.0056285 (PMC3567038; doi:10.1371/journal.pone.0056285)
Supplement: File S1 — Supplemental tables. (DOC) [file pone.0056285.s007.doc]

**Table S1. Composition of overall social sector expenditure by year across Indian states, 1997-2009.**

**Other social sector expenditure includes spending on housing, urban development, welfare of scheduled castes/tribes and other backward castes, labour and labour welfare, social security and welfare, relief on account of natural calamities, and other miscellaneous social sector categories.**

| **Year** | **Health (%)** | **Nutrition & water/sanitation (%)** | **Education (%)** | **Other social sector (%)** |
| --- | --- | --- | --- | --- |
| 1997 | 15.5 | 10.5 | 52.9 | 21.1 |
| 1998 | 15.0 | 10.1 | 54.8 | 20.1 |
| 1999 | 14.9 | 9.1 | 56.4 | 19.6 |
| 2000 | 14.4 | 9.6 | 55.0 | 21.0 |
| 2001 | 14.5 | 8.9 | 53.6 | 23.0 |
| 2002 | 14.1 | 9.2 | 52.5 | 24.2 |
| 2003 | 13.7 | 9.5 | 50.4 | 26.3 |
| 2004 | 13.5 | 10.1 | 50.2 | 26.2 |
| 2005 | 13.6 | 10.4 | 49.4 | 26.5 |
| 2006 | 13.3 | 10.1 | 48.1 | 28.5 |
| 2007 | 12.8 | 10.7 | 46.0 | 30.5 |
| 2008 | 12.2 | 10.2 | 44.3 | 33.2 |
| 2009 | 12.3 | 9.5 | 44.9 | 33.3 |

**Table S2. Correlations between components of social sector expenditure**

All expenditure is in log per-capita terms averaged for the five years ending in the current year.

|  | **Health** | **Nutrition & water/sanitation** | **Health-related** | **Education** | **Other social sector** | **Overall social sector** |
| --- | --- | --- | --- | --- | --- | --- |
| **Health** | 1 |  |  |  |  |  |
| **Nutrition & water/sanitation** | 0.592 | 1 |  |  |  |  |
| **Health-related** | 0.9004 | 0.8768 | 1 |  |  |  |
| **Education** | 0.8701 | 0.6427 | 0.8472 | 1 |  |  |
| **Other social sector** | 0.6937 | 0.6616 | 0.7611 | 0.6723 | 1 |  |
| **Overall social sector** | 0.9038 | 0.7857 | 0.9477 | 0.9411 | 0.8518 | 1 |
