# Supplementary material for: Social Sector Expenditure and Child Mortality in India: A State-Level Analysis from 1997 to 2009
Source: PLoS One. 2013 Feb 7;8(2):e56285. doi: 10.1371/journal.pone.0056285 (PMC3567038; doi:10.1371/journal.pone.0056285)

**Random intercepts**

The random intercepts by state and year in our models capture variation in mortality and death rates across states and years that is not accounted for by expenditure and poverty rates. Figures A and B below show the state and year random intercepts from models regressing the log of deaths rates at age 1-4 in boys and girls on log of per-capita overall social sector expenditure and poverty rates (i.e., the main model). From the scales in these figures, it is clear that residual variation in death rates is much larger across states than across years. Given the large mortality differentials and cultural, demographic, and economic differences between Indian states, this result is to be expected.

The state random intercepts show that, after controlling for overall social sector expenditure and poverty rates, predicted death rates in states like Kerala and Tamil Nadu are lower than the average death rate over all observations. On the other hand, the predicted death rates in Haryana, Rajasthan, Punjab, and Gujarat are higher than average. As Punjab and Haryana have lower poverty rates than most states (see Figure S2), these relatively wealthy states are doing quite poorly in terms of mortality in girls aged 1-4; even after taking their poverty advantage into account, death rates in these states are higher than average. Conversely, Bihar, a state with high poverty, has lower-than-average mortality in both boys and girls aged 1-4 after controlling for overall social sector expenditure and poverty rates.

**Figure A. State random intercepts from the model regressing death rates at ages 1-4 years on per-capita overall social sector expenditure and poverty rates.**

The random intercepts have been transformed from the scale of log death rates to the scale of death rates and centered at zero.


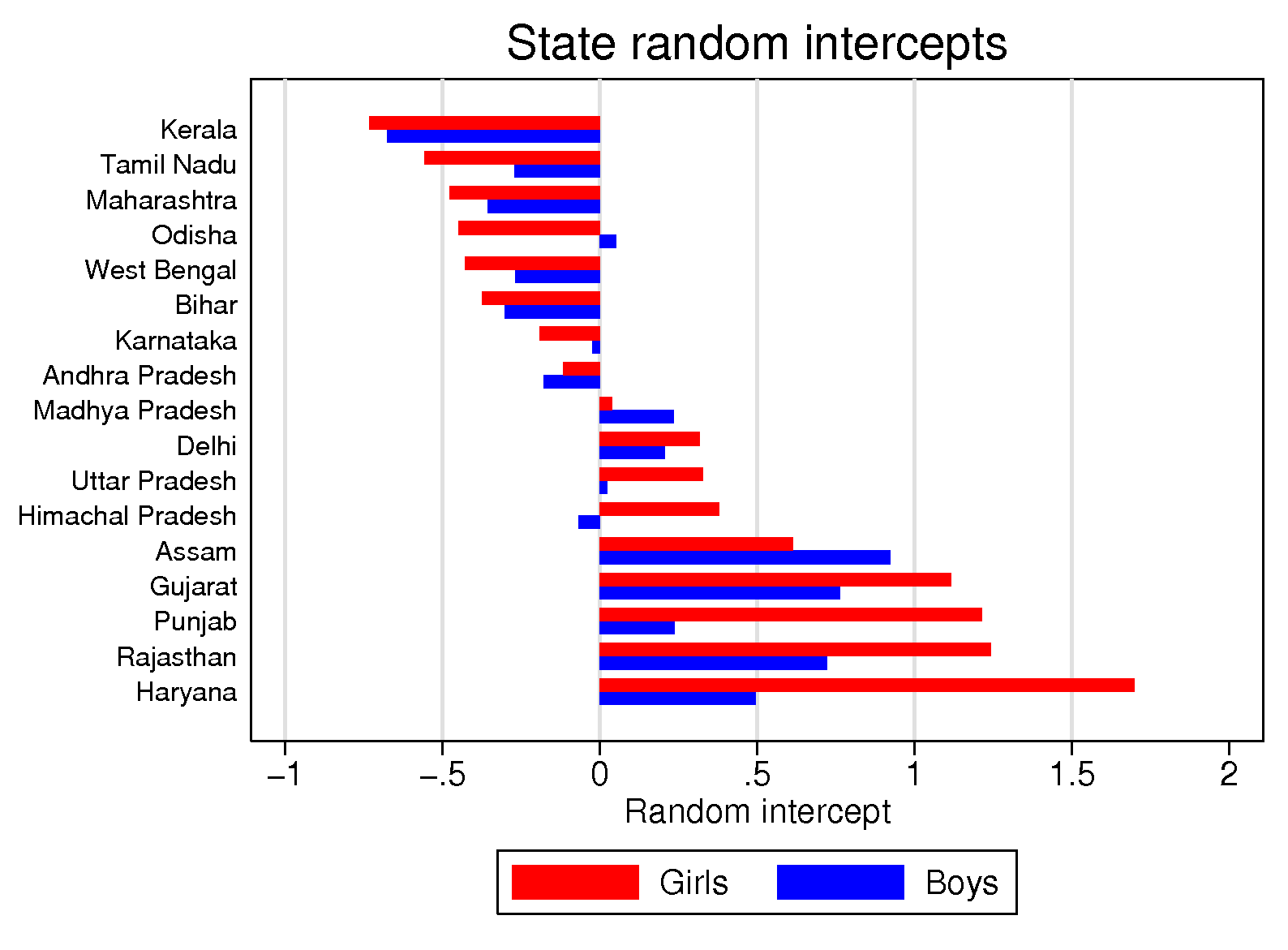


**Figure B. Year random intercepts from the model regressing death rates at ages 1-4 years on per-capita overall social sector expenditure and poverty rates.**

The random intercepts have been transformed from the scale of log death rates to the scale of death rates and centered at zero.


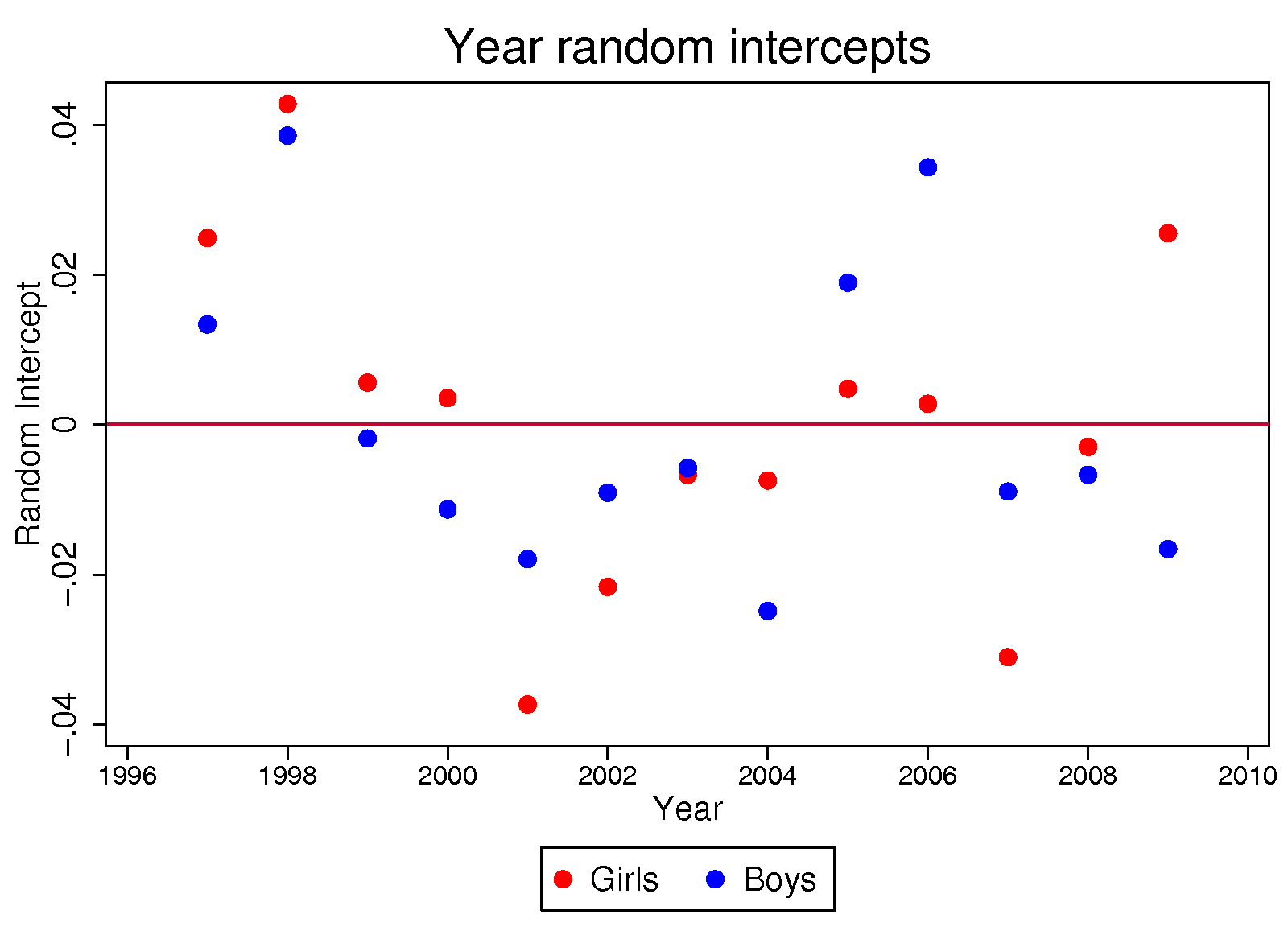

Supplement: File S2 — Random intercepts. (DOC) [file pone.0056285.s008.doc]
